# Can molecular dynamics simulations improve the structural accuracy and virtual screening performance of GPCR models?

**DOI:** 10.1371/journal.pcbi.1008936

**Published:** 2021-05-13

**Authors:** Jon Kapla, Ismael Rodríguez-Espigares, Flavio Ballante, Jana Selent, Jens Carlsson

**Affiliations:** 1 Science for Life Laboratory, Department of Cell and Molecular Biology, Uppsala University, Uppsala, Sweden; 2 Research Programme on Biomedical Informatics (GRIB), Department of Experimental and Health Sciences of Pompeu Fabra University (UPF), Hospital del Mar Medical Research Institute (IMIM), Barcelona, Spain; Icahn School of Medicine at Mount Sinai, UNITED STATES

## Abstract

The determination of G protein-coupled receptor (GPCR) structures at atomic resolution has improved understanding of cellular signaling and will accelerate the development of new drug candidates. However, experimental structures still remain unavailable for a majority of the GPCR family. GPCR structures and their interactions with ligands can also be modelled computationally, but such predictions have limited accuracy. In this work, we explored if molecular dynamics (MD) simulations could be used to refine the accuracy of *in silico* models of receptor-ligand complexes that were submitted to a community-wide assessment of GPCR structure prediction (GPCR Dock). Two simulation protocols were used to refine 30 models of the D_3_ dopamine receptor (D_3_R) in complex with an antagonist. Close to 60 μs of simulation time was generated and the resulting MD refined models were compared to a D_3_R crystal structure. In the MD simulations, the receptor models generally drifted further away from the crystal structure conformation. However, MD refinement was able to improve the accuracy of the ligand binding mode. The best refinement protocol improved agreement with the experimentally observed ligand binding mode for a majority of the models. Receptor structures with improved virtual screening performance, which was assessed by molecular docking of ligands and decoys, could also be identified among the MD refined models. Application of weak restraints to the transmembrane helixes in the MD simulations further improved predictions of the ligand binding mode and second extracellular loop. These results provide guidelines for application of MD refinement in prediction of GPCR-ligand complexes and directions for further method development.

## Author summary

The determination of detailed molecular structures of proteins is important to understand how they function and is an essential component of many drug discovery campaigns. Unfortunately, experimental structures are lacking for a large number of proteins and will be challenging to determine. One approach to solve this problem is to generate computer models. However, current modeling methods are not very accurate, and the predicted structures will contain errors. Consequently, there is a need for new approaches to increase the structural accuracy of protein models. In this work, we investigated if atomistic simulations can be used to refine models of a G protein-coupled receptor (GPCR) in complex with a drug-like ligand. GPCRs constitute a large family of membrane proteins which are important drug targets. Based on models of a dopamine receptor, our main finding is that refinement using molecular dynamics simulations can improve predictions of the binding mode of a ligand. Our results suggest that simulation-refined models of GPCR-ligand complexes can contribute to accelerating structure-based discovery and optimization of novel drug candidates.

## Introduction

Three-dimensional structures of proteins have contributed to detailed knowledge about biological processes at the molecular level. During the last two decades, advances in experimental techniques such as X-ray crystallography, NMR, and cryo-electron microscopy led to the determination of almost 170,000 atomic resolution structures [1]. However, the number of solved structures is still many orders of magnitude smaller than the 195 million sequence entries in UniProt [2]. Computational structure prediction could bridge this gap, but models with near experimental quality are required to study structure-function relationships and enable rational drug design.

Numerous computational approaches for protein structure prediction have been developed. A driving force of method development has been the Critical Assessment of protein Structure Prediction (CASP) experiments. The CASP assessments have challenged the research community to predict the 3D structures of proteins based on amino acid sequences. Models submitted to CASP are compared with experimentally determined structures, which are not available to participants during the assessment. The results enable comparisons of the performance of available methods and thereby benchmark the progress in the field. Protein structure prediction methods can broadly be divided into two classes, template-based (*e*.*g*. homology modeling and fold recognition) and *ab initio* (*e*.*g*. fragment assembly and physics-based methods, also known as template-free modeling) [3]. Both template-based and *ab initio* models will contain errors (*e*.*g*. in secondary structure, side chain packing, and loop regions) and need to be improved to be useful in applications that are sensitive to molecular details. For this reason, there is an increasing interest in methods that can enhance model quality. However, the CASP assessments have demonstrated that model refinement is very challenging. In CASP8, 70% of the participants in an evaluation of protein structure refinement failed to improve agreement with experimental data compared to initial models [4]. In the last decade, access to more computational power led to an increase of the use of physics-based methods such as molecular dynamics (MD) simulations and force fields in protein structure prediction [5–9]. Whereas physics-based methods initially had very limited success in CASP, performance has improved in recent assessments. In fact, MD simulation with physics-based force fields was used by eight out of the ten top-performing refinement methods in the recent CASP12 and all of these yielded an average improvement of structural accuracy [10]. Consistent improvement of model quality with physics-based techniques could have a major impact on studies of protein function and enhance the predictive power of structure-based drug design.

Two research areas that have generally not been evaluated by the CASP assessments are prediction of membrane protein structures and refinement of protein-drug complexes. As many membrane proteins (*e*.*g*. ion channels and G protein-coupled receptors) are therapeutically relevant [11,12] and structural coverage is still relatively limited for this group, there is a great need for accurate modeling approaches. The utility of protein models in rational drug design will be dependent on if structures of complexes with ligands can be predicted. The three GPCR dock assessments (GPCR Dock 2008, 2010, and 2013) [13–15] challenged the research community to predict structures of G protein-coupled receptors (GPCRs) in complex with ligands. The results of the first assessment in 2008, which focused on predicting the structure of the A_2A_ adenosine receptor in complex with an antagonist ligand, were discouraging. The vast majority of the participating research groups (93%) failed to predict the ligand binding mode accurately (RMSD > 3 Å). As expected, the best predictions of the receptor structures in the three GPCR Dock assessments were obtained with homology modeling and model accuracy was correlated with how closely related the target was to available GPCR crystal structures. However, even if templates with high sequence identity were available in the two last assessments, prediction of ligand binding modes remained challenging. In fact, around 60%, 80%, and 70% of the research groups failed to model the ligand binding mode accurately (RMSD > 3 Å) for the D_3_ dopamine, 5-HT_1B_ serotonin, and 5-HT_2B_ serotonin receptors, respectively. In the GPCR Dock assessments, prediction of the receptor-ligand complexes was typically performed based on a static homology model with no additional refinement steps. Only a few of the participating research groups used MD refinement to generate the models (based on supporting methods from the assessment participants [13–15]) and the impact of using simulations on the structural accuracy of the receptor-ligand complex was not evaluated.

In this study, we assessed the use of MD refinement to improve structural models of GPCRs, ligand binding modes, and virtual screening performance. Simulations of the D_3_ dopamine receptor (D_3_R) in complex with the ligand eticlopride, which was one of the targets of GPCR Dock 2010 [14], were performed for 30 of the models submitted to the assessment. Two protocols based on different force fields were used to generate a total MD simulation time of close to 60 μs. Snapshots from the simulation trajectories were compared to the crystal structure of the D_3_R-eticlopride complex [16] to determine the accuracy of the transmembrane (TM) region, loops, binding site, and ligand binding mode. Strategies for improving the MD refinement protocols by using restraints in the simulations were also explored. Advantages and drawbacks of using MD refined GPCR-ligand complexes were analyzed based on comparisons to the models used as starting structure, simulations of the crystal structure, and the virtual screening performance of the models.

## Results

### Benchmarking set, simulation protocols, and assessment criteria

A set of 30 models of the D_3_R in complex with the antagonist eticlopride was obtained from the GPCR Dock 2010 assessment [14]. The models represented different levels of accuracy of the receptor-ligand complex and originated from 25 participants. The systems were set up for MD simulations using two different protocols. In the first protocol, the receptor and ligand were modelled using OPLS-AA force field parameters [17], and the simulations were performed in GROMACS [18]. We refer to this protocol as OPLS. In the second protocol, the protein and ligand were modeled with CHARMM force field parameters [19,20], and simulations were carried out in ACEMD [21]. We refer to this protocol as CHARMM. In each protocol, the receptor-ligand complexes were embedded in a bilayer using 1-palmitoyl-2-oleoyl-*sn*-glycero-3-phosphocholine (POPC) lipids and solvated with water molecules. A more detailed comparison of the OPLS and CHARMM protocols is summarized in Table A in S1 Appendix. After a short equilibration, three independent simulations of 100 ns were carried out for each system and protocol. For comparison, three simulations of the same length were also carried out for the crystal structure of D_3_R in complex with the same ligand using both protocols (PDB code: 3PBL) [16].

**Fig 1:**
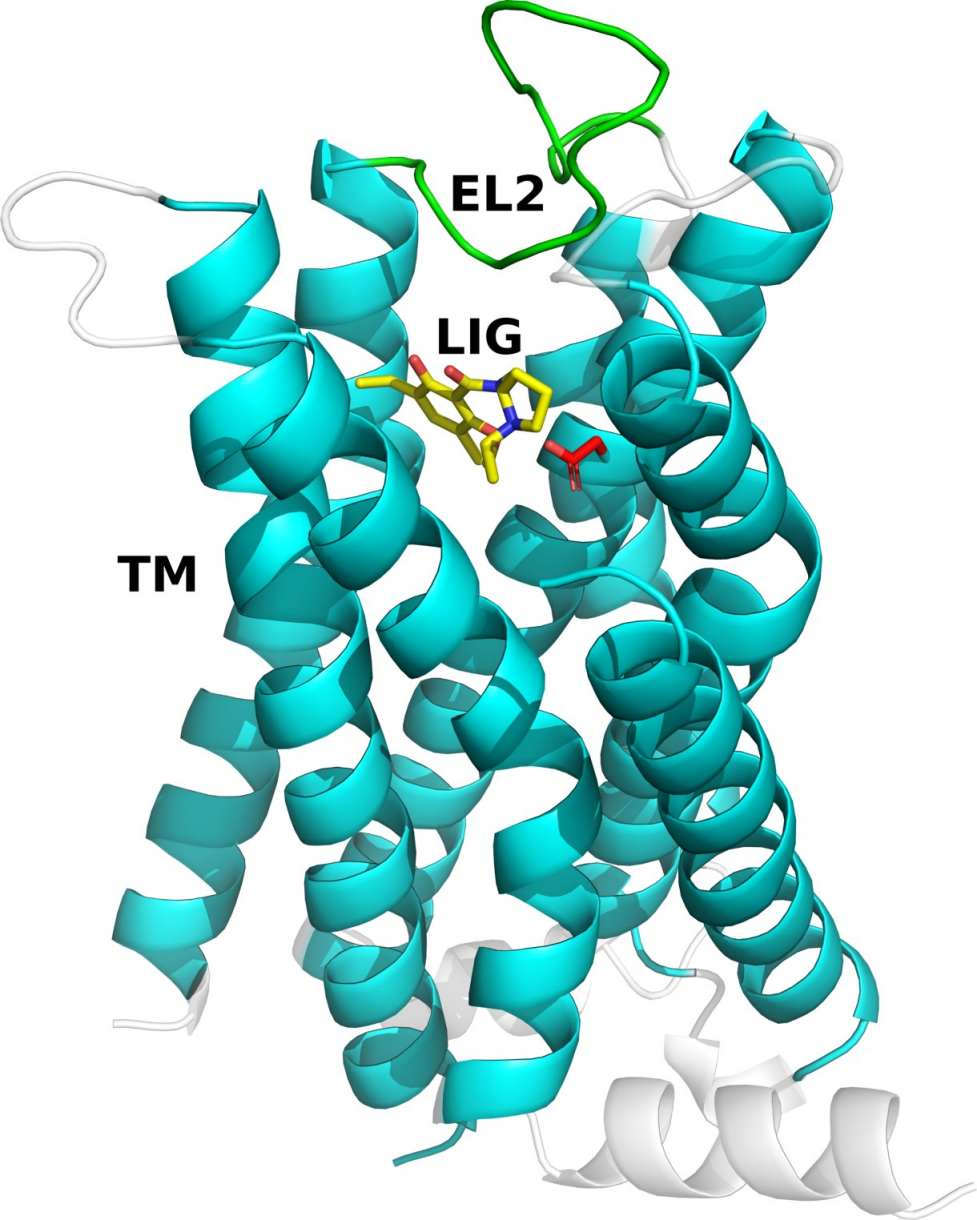
Structure of the D_3_R in complex with eticlopride. The structure of the D_3_R is shown as cartoons and the ligand as sticks (PDB code: 3PBL). The D_3_R models were assessed based on the TM region (cyan), EL2 (green) and ligand (LIG, yellow). The ligand eticlopride forms a salt bridge with Asp110 (red sticks).

To assess the accuracy of the MD refined models, we primarily compared the backbone of the transmembrane (TM) region and the second extracellular loop (EL2) as well as ligand heavy atoms (LIG) to the D_3_R crystal structure (Fig 1). These three selections were also the main focus of the GPCR Dock assessments [13–15]. The TM RMSD from the crystal structure is a measure of the prediction accuracy of the overall receptor structure, but does not reflect the accuracy of the loop regions or ligand binding mode. The EL2 is difficult to model by homology as these sequences are poorly conserved, but this region plays a crucial role in ligand binding for many GPCRs. In the case of the D_3_R, EL2 interacts directly with the co-crystallized ligand eticlopride and modeling of this region was one of the major challenges in the GPCR Dock assessments [13–15]. In order to assess the value of the models in rational drug design, particular focus was also put on the ligand and binding site.

For each model and protocol, ~1500 snapshots from the three simulation trajectories were aligned to the backbone atoms of the TM region and clustered based on ligand RMSD. The centroids of the five largest clusters of the ligand were identified and compared to the crystal structure. This approach mimicked the submission rules of the GPCR Dock assessments [13–15] in which the participants were allowed to submit a maximum of five models. We hence investigated if the MD refinement protocols could have improved the performance of the research groups participating in the assessment. In the GPCR Dock assessment, an adaptive alignment method was used to align the models to the crystal structure [22]. We used a standard rotational and translational alignment based on the minimization of RMSD from the reference structure. Our calculated RMSD values for the initial models are in excellent agreement with those reported GPCR Dock 2010 except in one case (model M-30), which was one of the least accurate models and ambiguous to align (R^2^ ≥ 0.98, if model M-30 is treated as an outlier, Fig A in S1 Appendix) [23]. The analysis of the results was primarily focused on the median change in RMSD from the crystal structure (*i*.*e*. we compared the third best centroid RMSD for each model to the initial RMSD value) and the best result obtained from the five centroids.

### The TM region of MD refined models drifts further away from the crystal structure conformation

**Fig 2:**
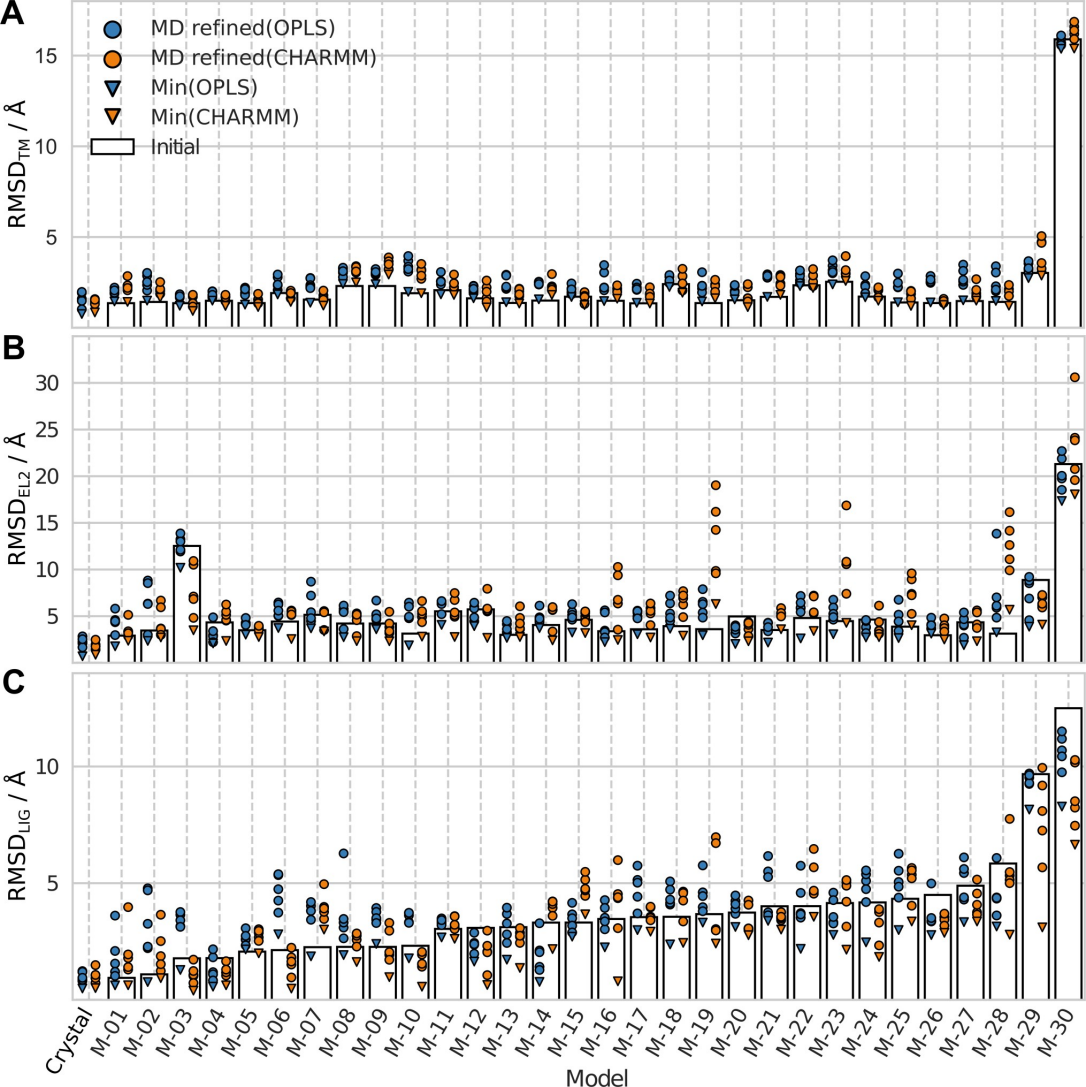
RMSD from crystal structure for initial and MD refined structures. RMSDs for the initial structures (bars) and the five MD refined models (cluster centroids) from the OPLS (blue circles) and CHARMM (orange circles) protocols. The minimum RMSD values from the MD simulations are shown as triangles. The RMSD values were calculated for the (A) TM region, (B) EL2, and (C) ligand (LIG).

The overall structural accuracy of the MD refined models was evaluated based on the TM backbone RMSD (RMSD_TM_) from the crystal structure. Fig 2A shows the RMSD_TM_ values of each model prior to refinement and the centroids belonging to the five largest clusters from the MD simulations. A majority of the GPCR Dock models (22/30) had initial RMSD_TM_ < 2 Å. Six of the models had RMSD_TM_ values between 2 and 3 Å. One of the remaining two models had an RMSD_TM_ slightly higher than 3 Å and the worst model M-30 had a large RMSD_TM_ of 16 Å. Visual inspection of the initial models revealed that TM5, TM6 and TM7 showed the largest deviations from the D_3_R crystal structure in the TM region. The intracellular parts of TM5 and TM6 were generally shifted towards TM7. In the extracellular region, TM6 and TM7 were instead shifted toward TM5. Some of the models also showed deviations in TM1 close to the N-terminus, and in TM4 close to the second intracellular loop (IL2). These differences generally reflected limitations of the crystal structure templates used in modelling by the GPCR Dock participants.

Based on the RMSD_TM_ values of the MD refined models, the overall structure of the receptor models generally drifted further away from the crystal structure using both simulation protocols. Overall, 3% and 37% of the models had at least one MD refined structure with better RMSD_TM_ than the initial structure for the OPLS and CHARMM protocols, respectively (Table 1). The median difference between the RMSD_TM_ values of the MD refined and GPCR Dock models (ΔRMSD_TM_) was positive regardless of the initial quality of the GPCR Dock models (Table 2). In fact, analysis of all 1500 snapshots obtained for each model to identify the one that was most similar to the crystal structure showed that the median difference between the minimum and initial RMSD_TM_ was close to zero for the OPLS protocol and slightly negative for CHARMM. As the minimum RMSD_TM_ generally was close to the best centroid (Fig 2A), the lack of improvement in the TM region for the five selected MD refined structures was not due to the snapshot selection procedure. The main difference between the two protocols was that OPLS showed larger fluctuations in the TM-region than CHARMM (Fig B in S1 Appendix), which is consistent with previous comparisons of OPLS and CHARMM force fields for soluble proteins [24].

**Table 1:** Percentage of improved models after MD refinement.

ΔRMSD_LIG_ / Å^a^	OPLS / %	CHARMM / %	OPLS (restrained) / %^b^
< 0.0	50	73	60
< –0.5	30	57	37
< –1.0	17	30	23
**ΔRMSD**_**TM**_ **/ Å**^**a**^			
< 0.0	3	37	43
< –0.05	3	20	23
< –0.1	3	17	10
**ΔRMSD**_**EL2**_ **/ Å**^**a**^			
< 0.0	50	50	70
< –0.5	37	37	53
< –1.0	23	27	27

^a^ Calculated as the difference in RMSD between the best MD refined (selected from the centroids representing the five largest clusters for each model) and the initial structure (ΔRMSD = RMSD_MD refined_−RMSD_Initial model_).

^b^ Simulations were performed with restraints on C_α_ atoms in the TM region.

**Table 2:** Accuracy of the TM region, EL2 and ligand (LIG) after MD refinement based on the difference in RMSD compared to the initial model (ΔRMSD = RMSD_MD refined_−RMSD_Initial model_).

**OPLS protocol**					
**Model quality**^**a**^		**Selection**	**ΔRMSD/Å**^**b**^	**ΔRMSD**_**Best**_**/Å**^**c**^	**ΔRMSD**_**Min**_**/Å**^**d**^
**All**		**LIG**	0.54	–0.02	–0.90
		**TM**	0.71	0.51	0.01
		**EL2**	0.72	–0.06	–1.3
**Good**	0.0–2.5 Å	**LIG**	1.3	1.0	–0.34
	0.0–1.5 Å	**TM**	0.86	0.61	0.04
	0.0–4.0 Å	**EL2**	1.4	0.56	–0.65
**Medium**	2.5–5.0 Å	**LIG**	0.31	–0.43	–1.3
	1.5–2.5 Å	**TM**	0.52	0.33	–0.02
	4.0–5.0 Å	**EL2**	0.67	0.02	–1.5
**Bad**	> 5.0 Å	**LIG**	–1.5	–2.2	–2.7
	> 2.5 Å	**TM**	0.26	0.03	–0.28
	> 5.0 Å	**EL2**	0.19	–0.96	–2.1
**CHARMM protocol**					
**Model quality**^**a**^		**Selection**	**ΔRMSD/Å**^**b**^	**ΔRMSD**_**Best**_**/Å**^**c**^	**ΔRMSD**_**Min**_**/Å**^**d**^
**All**		**LIG**	–0.10	–0.63	–1.2
		**TM**	0.49	0.22	–0.14
		**EL2**	0.84	–0.02	–1.4
**Good**	0.0–2.5 Å	**LIG**	0.13	–0.27	–0.91
	0.0–1.5 Å	**TM**	0.53	0.34	–0.02
	0.0–4.0 Å	**EL2**	2.0	0.60	–0.45
**Medium**	2.5–5.0 Å	**LIG**	0.17	–0.60	–1.1
	1.5–2.5 Å	**TM**	0.37	–0.04	–0.26
	4.0–5.0 Å	**EL2**	0.80	–0.61	–1.9
**Bad**	> 5.0 Å	**LIG**	–1.6	–4.0	–5.8
	> 2.5 Å	**TM**	0.64	0.24	–0.14
	> 5.0 Å	**EL2**	–0.11	–1.7	–3.1

^a^ Based on the RMSD values of the GPCR Dock models.

^b^ Calculated based on the median of the centroids representing the five largest clusters from each MD refinement, *i*.*e*. the third best result of each MD refinement is used to calculate the ΔRMSD.

^c^ Calculated based on the best RMSD value obtained from the centroids representing the five largest clusters from each MD refinement.

^d^ Calculated based on the minimum RMSD identified in all 1500 snapshots generated for each model.

Analysis of the MD refined models showed that the largest fluctuations in the TM region were close to the loop regions, in particular close to EL3 (TM6 and 7), but also at EL1 (TM2 and TM3) and IL2 (TM3 and TM4). Some extent of unfolding was identified in both the OPLS and CHARMM simulations, *e*.*g*. at the N-terminal ends of TM4 and TM6. In the CHARMM simulations, unfolding of the C-terminal end of TM5 was observed for some, but not a majority, of the models. In this context, it should be noted that part of IL3, which connects TM5 and TM6, was replaced by T4-lysozyme in the D_3_R crystal structure, an insertion that facilitates crystallization. The models did not include this insertion and, as IL3 is too long to predict accurately, all simulations excluded this part of the sequence. Some of the fluctuations and partial unfolding that were observed in this region can likely be attributed to this sequence gap.

**Fig 3:**
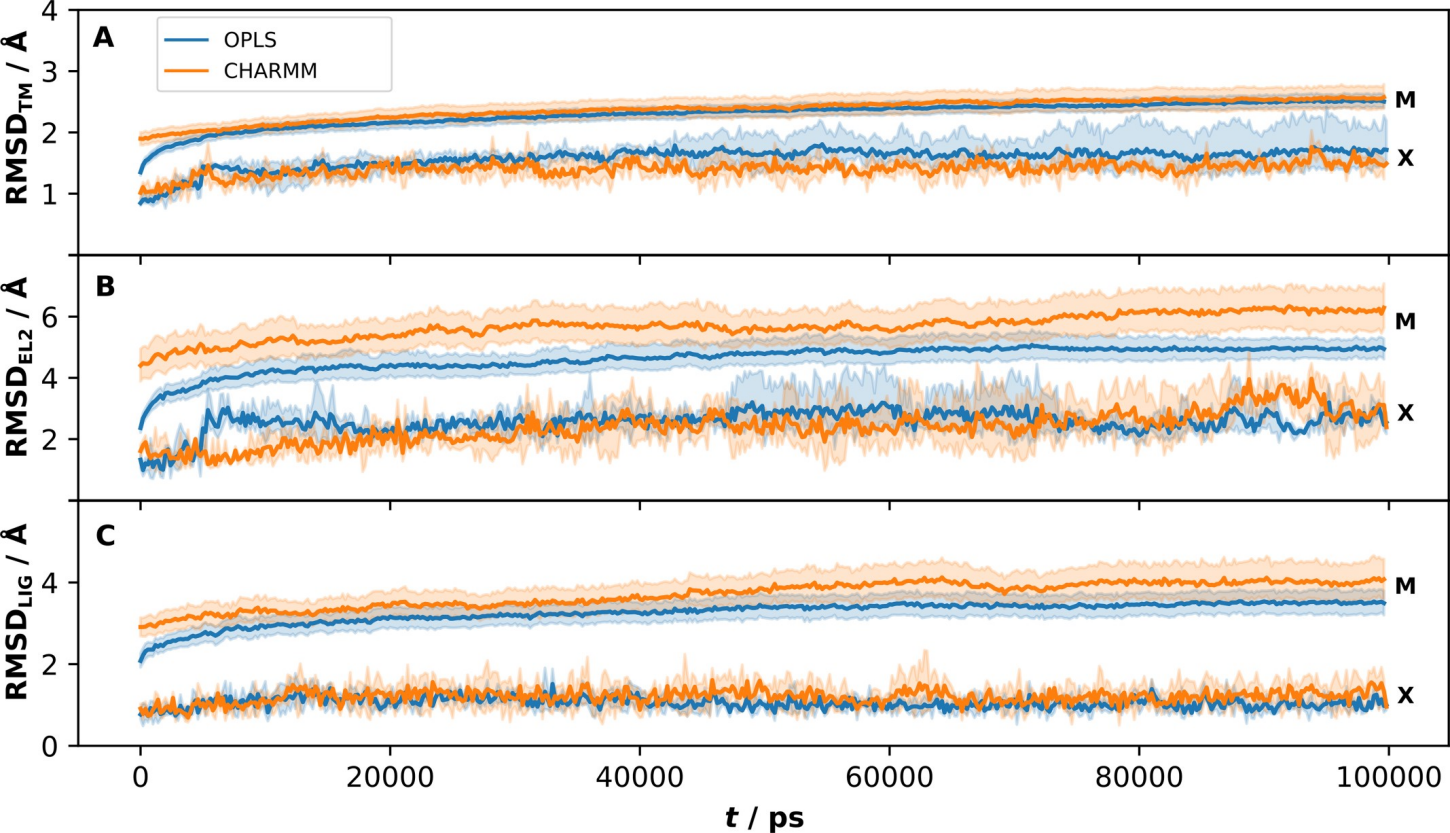
RMSD from the initial structure for the D_3_R models and crystal structure. Averages per time frame over all model (M) and the crystal structure (X) simulations for the OPLS (blue) and CHARMM (orange) protocols. The RMSD was calculated with the initial structure as reference for the (A) TM region, (B) EL2 and (C) ligand (LIG). The standard error at 95% confidence interval is indicated with paler colors.

**Fig 4:**
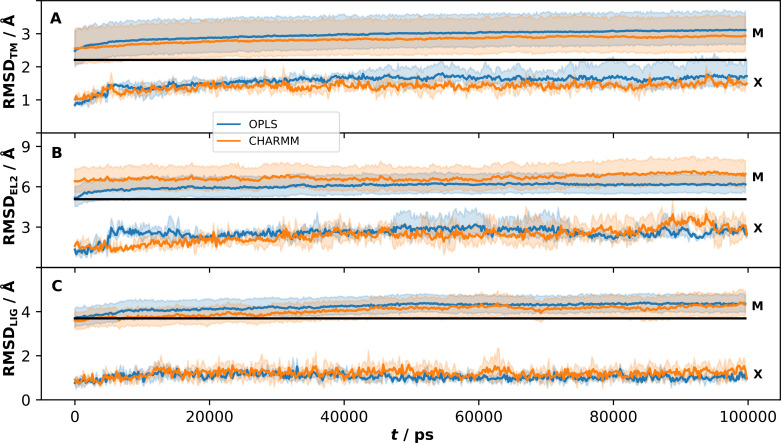
RMSD from the crystal structure for the D_3_R models and crystal structure simulations. Averages per time frame over all model (M) and the crystal structure (X) simulations for the OPLS (blue) and CHARMM (orange) protocols. The RMSD was calculated with the crystal structure as reference for the (A) TM region, (B) EL2 and (C) ligand (LIG). The standard error at 95% confidence interval is indicated with paler colors. The black horizontal line corresponds to the average deviation from the crystal structure for the initial models.

For comparison, MD simulations of the crystal structure of the D_3_R in complex with eticlopride were also performed and clustered with the same protocol as the models. In agreement with the results for the models, the TM region exhibited an RMSD increase as soon as the restraints were fully released and relaxed to an average RMSD_TM_ of 1.6 and 1.4 Å for the OPLS and CHARMM protocols, respectively. Similar initial changes in RMSD have been observed for simulations of soluble proteins in the Dynameomics database [25] and of a β_2_ adrenergic receptor crystal structure [26]. The crystal structure was clearly more stable than the models in all three simulation replicates and with both simulation protocols (Fig C in S1 Appendix). Visual inspection of the intracellular ends of TM5 and TM6 for the crystal structure simulations revealed no large deviations due to the missing IL3. This is in accordance with previous long simulations of the β_2_ adrenergic receptor, in which the inactive conformation of the receptor was found to be stable even if IL3 was not modelled explicitly [27]. In contrast, a few of the simulations based on models were unstable and the RMSD_TM_ continued to increase over the entire simulation length and this behaviour was independent of the simulation protocol (Fig C in S1 Appendix). To compare the behaviour of the crystal structure to the 30 models, we calculated the average RMSD_TM_ from the initial structure (Fig 3A) and the crystal structures (Fig 4A), resulting in 1500 averages at points along the 100 ns trajectory based on a total of 9 μs of simulation time per protocol. The average RMSD_TM_ for the crystal structure increased to values of ~1.5 Å during equilibration for both protocols and remained stable at this level during the simulation. For the models, the RMSD_TM_ from the initial coordinates increased to higher values > 2 Å, and there was a slight drift towards increased values after 100 ns. As the initial models deviated with up to 3 Å from the crystal structure, this would be the magnitude of structural changes that would be necessary to substantially improve structural accuracy. However, the RMSD_TM_ values calculated with the crystal structure as reference show that the models drift further away from this conformation. The initial average deviation was 2.1 Å and the MD refinement led to values of around 3.0 Å (Fig 4A). These results were independent of the simulation protocol.

To assess how the simulations affected the receptor structures, the initial and MD refined models were scored with two different methods that evaluate protein structure quality, Molprobity [28] and normalized DOPE [29,30] (n-DOPE). The Molprobity scores were consistently improved by MD refinement, which was due to a reduction of clashes in the initial models, whereas the n-DOPE scores generally were slightly worse (Fig D in S1 Appendix). There was no correlation between RMSD_TM_ and the quality scores. The structure quality scores of the MD refined structures were generally better for CHARMM than for the OPLS protocol.

### For 50% of the models, MD refinement leads to at least one structure with a more accurate second extracellular loop

The RMSD of EL2 (RMSD_EL2_) for the initial and the MD refined models are shown in Fig 2B. The RMSD_EL2_ exhibited substantial fluctuations over the simulations, resulting in a diverse set of loop conformations for each receptor model. The median ΔRMSD_EL2_ values were positive for both protocols, and hence, there was an overall drift away from the crystal structure in the EL2 region (Table 2). The OPLS protocol (median of 0.72 Å) performed slightly better than CHARMM (median of 0.84 Å) and this was also evident from the average values calculated along the MD trajectory (Fig 4B). If only the best MD refined structure for each model was considered, 50% of the EL2 conformations were improved (Table 1). In this case, both protocols were able to improve the prediction of EL2 with median ΔRMSD_EL2_ values of −0.06 Å (OPLS) and −0.02 Å (CHARMM). In agreement with the results for the TM region, the average RMSD_EL2_ for the crystal structure increased, but the overall fold of the loop was maintained, and the deviation from the initial structure was larger in simulations of the GPCR Dock models (Fig 3B).

**Fig 5:**
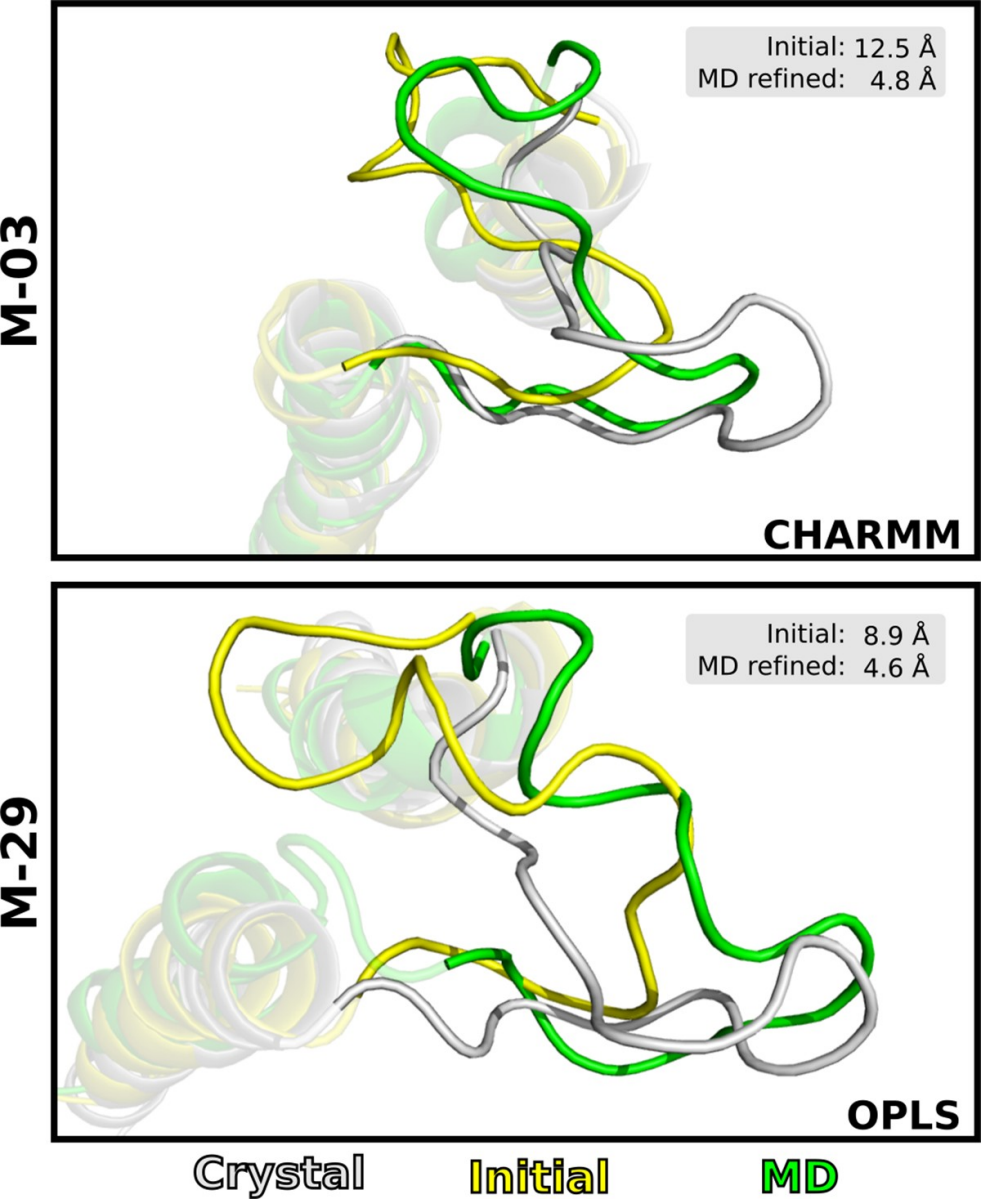
MD refinement of loop region. Examples of successful refinements of the EL2 region of models M-03 and M-29. TM4-5 and EL2 are shown as cartoons for the initial model (yellow), MD refined model (green) and the D_3_R crystal structure (grey).

Inspection of MD refined EL2 structures showed that substantial improvements of the EL2 region could be obtained (Fig 5). The model with largest decrease of RMSD_EL2_ was M-03. For the CHARMM protocol, a reduction of RMSD_EL2_ from 12.5 Å to 4.8 Å was achieved. The model M-29 had an initial RMSD_EL2_ value of 8.9 Å. Both simulation protocols were able to refine the loop region with improvements of –4.3 Å and –3.0 Å for OPLS and CHARMM, respectively. Notably, the EL2 region that is part of the orthosteric binding site improved for both M-29 and M-03.

### The CHARMM protocol can refine the ligand pose

The ligand heavy atom RMSD (RMSD_LIG_) of the GPCR Dock models and the MD refined structures are shown in Fig 2C. It was clear that MD refinement could improve the ligand pose and the best result was obtained with the CHARMM protocol. The median ΔRMSD_LIG_ (*i*.*e*. the median of the third best improvement of the 30 MD refinements) did not improve compared to the unrefined model for OPLS (median ΔRMSD_LIG_ = 0.54 Å), but showed improvement for CHARMM (median ΔRMSD_LIG_ = –0.10 Å). If all MD refined models were considered, 51% of the structures obtained with the CHARMM protocol improved and the corresponding value for OPLS was 31%. If only the best of the five MD refined models (centroids of the five largest clusters, Fig 2C) were included, improvements of the RMSD_LIG_ were obtained in 50% and 73% of the simulations for the OPLS (median ΔRMSD_LIG_ = –0.02 Å) and CHARMM (median ΔRMSD_LIG_ = –0.63 Å) protocols, respectively (Table 2). If we identified large improvements of >1 Å, these numbers were reduced to 17% (OPLS) and 30% (CHARMM). The minimum RMSD_LIG_ among the 1500 simulation snapshots, which represents the best possible result that could have been achieved, was also identified for each trajectory. It was possible to find a minimum RMSD_LIG_ that was better in most cases and the median improvements were –0.9 and –1.2 Å for the OPLS and CHARMM protocols, respectively. As these snapshots were not identified by the clustering, such large improvements cannot be expected in applications of the MD refinement protocols to predict the (unknown) binding mode of a ligand. However, it was interesting that the relative performance of the protocols was the same for the median, best, and minimum ΔRMSD_LIG_ values. In all comparisons, CHARMM performed better than OPLS and this result was also clear from the average RMSD_LIG_ values calculated along the MD trajectory (Fig 4C). The CHARMM protocol also performed better for the binding site structure. Binding site side chain RMSD values (RMSD_SC_) were improved for 38% and 19% of the models with the CHARMM and OPLS protocols, respectively (Table B in S1 Appendix), and larger improvements were also obtained for the best models from the CHARMM simulations (Table C in S1 Appendix).

The performance of the MD refinement protocols was dependent on the initial RMSD_LIG_ of the models (Table 2). In a few cases within the RMSD range 0–2.5 Å (*e*.*g*. models M-03, M-06, and M-10), the models simulated with OPLS drifted further away from the crystal structure, whereas the CHARMM simulations led to improvements of both the ligand and binding site RMSD values (Fig 2C and Table C in S1 Appendix). The median best centroid ΔRMSD_LIG_ for these models was substantially worse for OPLS than for CHARMM (1.0 and –0.27 Å for OPLS and CHARMM, respectively). In the medium range of initial RMSD_LIG_ values (2.5–5 Å), both protocols improved the models and the median best centroid ΔRMSD_LIG_ was –0.43 and –0.60 Å for OPLS and CHARMM, respectively. The models that initially showed a ligand RMSD ≳ 5 Å were improved by MD refinement with median best centroid ΔRMSD_LIG_ values below –2.0 Å for both protocols.

Similar to the TM and EL2 region, the ligand pose was more stable in the simulations based on the crystal structure than the GPCR Dock models (Fig 3C and Fig E in S1 Appendix). The average RMSD_LIG_ values were 1.1 and 1.2 Å for the OPLS and CHARMM protocols, respectively. The MD refined complexes were in excellent agreement with the experimental ligand binding mode (Fig F in S1 Appendix, RMSD_LIG_ of 0.81 and 0.73 Å for OPLS and CHARMM, respectively). The RMSD_LIG_ values for the models increased to 3–4 Å during the first 50 ns and then stabilized around this value (Fig 3C). Although the average RMSD_LIG_ values with the crystal structure as reference were lowest in the first 10 ns (Fig 4C and Fig G in S1 Appendix), it should be noted that the best MD refined models from the clustering were approximately uniformly distributed over the 100 ns simulation (Fig G in S1 Appendix).

To develop a protocol that would be useful in a real case scenario (i.e. when the ligand binding mode is unknown), we investigated how likely it was to obtain a better ligand pose if only the centroid from the largest cluster was considered for the CHARMM protocol. In this case, we obtained better poses in 57% of the refinements. In practice, it may be relevant to inspect several of the largest clusters from the MD refinement to increase the probability of generating an improved binding mode (Table D in S1 Appendix). The optimal choice was to include the three largest clusters, which resulted in an improved binding mode in 70% of the MD refinements. Further increasing the number of considered centroids only led to a small increase of the probability of generating an improved ligand binding mode (73% for both four and five clusters).

**Fig 6:**
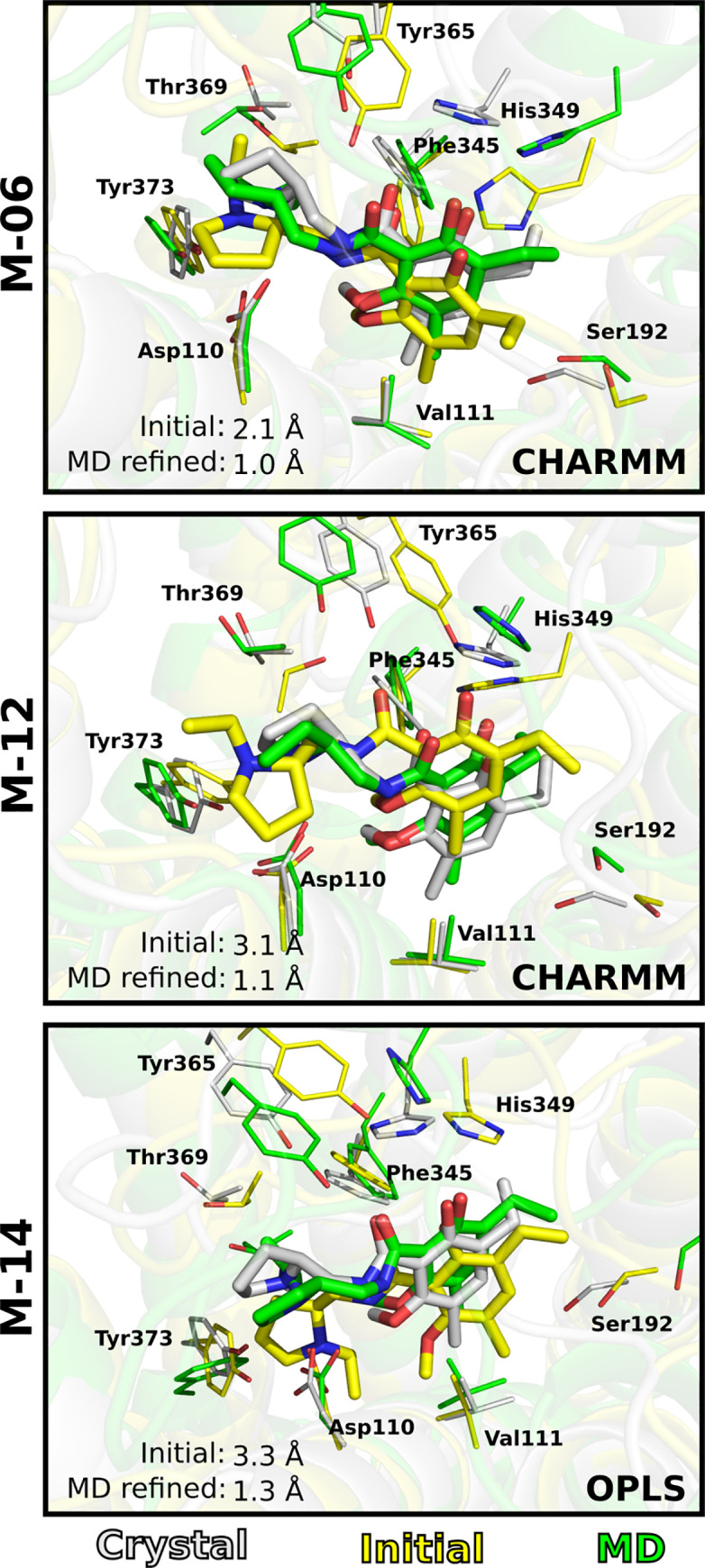
MD refinement of ligand binding mode. Examples of MD refined binding modes for models M-06, M-12 and M-14. One of the five cluster centroids extracted from the MD simulations is shown. The ligand and key residues are shown as sticks. The initial (yellow), MD refined (green) and crystal (grey) structures are shown as cartoons.

Fig 6 shows examples of improved ligand poses among the MD refined structures (centroids representing the five largest clusters, Fig 2C). The initial pose of model M-06 was relatively accurate (RMSD_LIG_ = 2.1 Å) and the CHARMM protocol was able to further refine the binding mode to 1.0 Å. The binding site RMSD also improved (ΔRMSD_SC_ = –0.2 Å). In particular, the side chains of Asp110 (–0.70 Å), Ile183 (–1 Å), Ser192 (–1.3 Å), Phe345 (–0.86 Å), and Tyr365 (–0.20 Å) agreed better with the crystal structure after refinement. For model M-12 the initial RMSD was 3.1 Å. After refinement, the ligand formed the important salt bridge with Asp110, resulting in an RMSD_LIG_ of 1.1 Å for the CHARMM protocol. In addition, the binding site was also improved by the MD refinement (ΔRMSD_SC_ = –0.86 Å). Large improvements were obtained for Ser192 (–1.1 Å), His349 (–2.1 Å), Tyr365 (–1.3 Å), and Thr369 (–2.0 Å). In the case of model M-14, the OPLS protocol successfully refined the initial pose. The initial RMSD was 3.3 Å, and after the OPLS protocol, the best structure was refined to 1.3 Å with a clearly improved binding mode. The OPLS simulations did not improve the accuracy of the binding site structure for M-14 (ΔRMSD_SC_ = 0.96 Å). For example, the RMSD_SC_ increased for Tyr365 and Thr369 by 0.2 Å and 1.6 Å, respectively, due to movements in the top of TM helix 7. Despite the overall reduction of binding site accuracy, His349 and Val111 were improved by –0.63 Å and –0.40 Å, respectively.

### What is the influence of the quality of the receptor model on refinement of the ligand pose?

To assess the influence of the quality of the receptor model on the possibility to refine a ligand binding mode, we used molecular docking to generate multiple poses in the D_3_R crystal structure and then refined these complexes with both MD protocols. Eticlopride was docked to the crystal structure and ten diverse poses (XD-01–XD-10) with RMSD values ranging from 1.0 to 7.0 Å from the crystal pose were selected. These ten complexes were then refined using the two MD protocols based on three 500 ns trajectories for each model.

**Fig 7:**
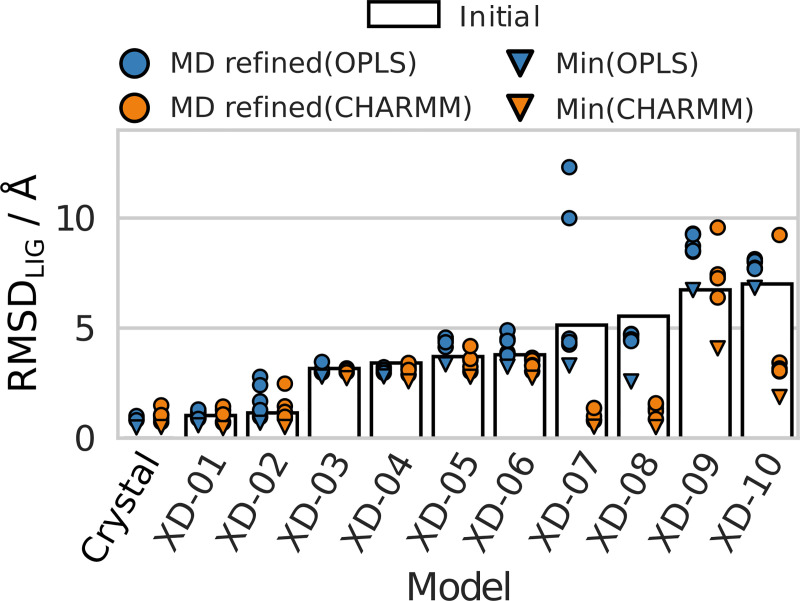
RMSD_LIG_ for the MD refined D_3_R crystal structure with diverse eticlopride binding modes generated with molecular docking. RMSDs for the initial structures (bars) and the five MD refined models from the OPLS (blue circles) and CHARMM (orange circles) protocols are shown. The minimum RMSD values from the MD simulations are shown as triangles. Data from simulations of the crystal structure are included as reference.

**Fig 8:**
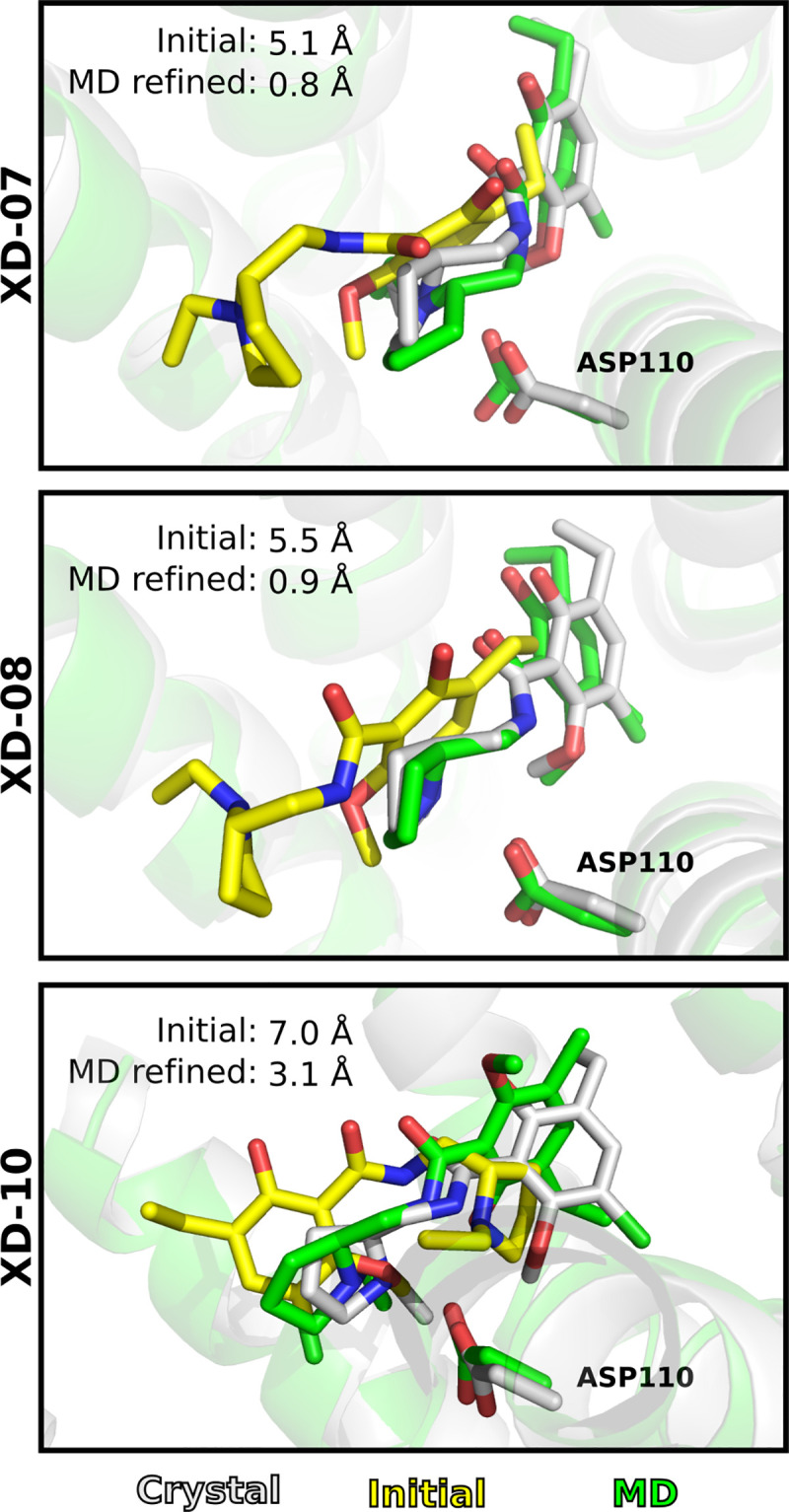
MD refinement of D_3_R crystal structure with binding modes of eticlopride generated by molecular docking. The ligand is shown as sticks. The crystal (grey), initial (yellow) and MD refined (green) receptor structures are shown as cartoons. The three models were refined with the CHARMM protocol.

Six models had initial RMSD_LIG_ values < 5 Å. After the MD refinement, the same binding modes were generally maintained (Fig 7) and this result was most pronounced for the CHARMM protocol. With the OPLS protocol, the RMSD increased more than for the CHARMM protocol for three out of the six models, but the best centroid still remained close to the initial pose. The four models with an initial RMSD > 5 Å showed larger variations in the results. For three of the models (XD-07, XD-08, and XD-10) large improvements of up to 4.6 Å were obtained with the CHARMM protocol (Fig 8). The initial poses of models XD-07 and XD-08 are similar (RMSD_LIG_ 5.1 and 5.5 Å) and the refinement involved a translation of the ligand in one dimension. In the initial structure for model XD-10 (RMSD_LIG_ 7.0 Å), the ligand was rotated ~180 degrees compared to the crystal structure, and the refinement involved both rotational and translational motion of the ligand. Interestingly, the improvements were generally larger than for the GPCR Dock models with similar initial accuracy of the binding mode.

### Restraints on the TM region improve loop and ligand refinement

The TM region tended to drift away from the reference during the unrestrained MD simulations. One approach to address this problem is to impose distance or position restraints to limit the search to conformations that are close to the initial structure. To examine the effect of restraints, the models and the crystal structure were simulated with weak C_α_ position restraints in the TM region with the OPLS protocol.

**Fig 9:**
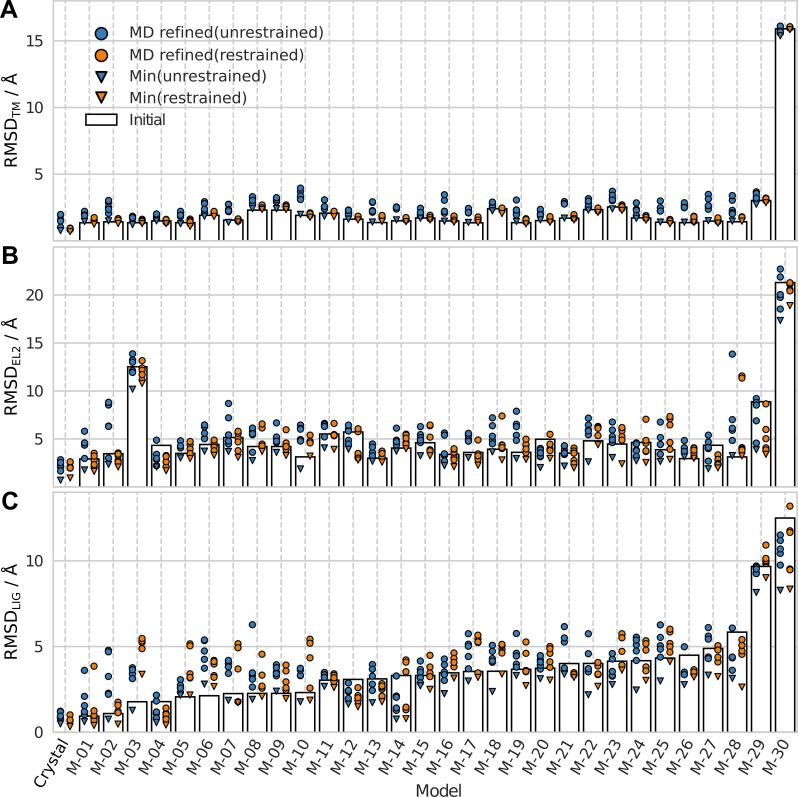
Comparison of MD refinement with restrained and unrestrained TM region. The initial (bars) and MD refined (circles) structures are shown for the (A) TM region, (B) EL2, and (C) ligand (LIG). Data from simulations with unrestrained (blue) and restrained (orange) TM region using the OPLS protocol are shown.

As expected, the restraints stabilized the TM region, which remained close to the initial structure, and there was a smaller variation between the cluster centroids compared to the unrestrained simulations (Fig 9A). The fraction of MD refinements resulting in a model with better RMSD_TM_ increased from 3% in the unrestrained simulations to 43% in the restrained variant (Table 1). The improvement from using restraints was also clear for the differences in ΔRMSD_TM_ values (Fig H in S1 Appendix). EL2 (Fig 9B) and the ligand (Fig 9C) were not restrained in the simulation, but the more rigid TM region improved the results compared to the unrestrained protocol. In 70% and 60% of the cases, at least one MD refined model led to improvements of the EL2 and ligand binding mode, respectively (Table 1). The corresponding values were 50% (EL2) and 50% (ligand) for the unrestrained simulations. The percentage of the models that had large improvements in EL2 or the ligand binding mode (> 1 Å) was also slightly higher (27% and 23%) compared to the unrestrained protocol (23% and 17%). The improvements were also evident from the median change of RMSD values compared to the initial models, in particular for the best refinements of each model (Table E in S1 Appendix).

### MD refinement can improve virtual screening performance

A set of 475 known D_3_R ligands and ~34,000 property-matched decoys from the DUD-E database [31] was used to assess virtual screening performance. The compounds were docked to the crystal structure, 30 GPCR Dock models, and the 155 MD refined centroids using DOCK3.7 [32]. The ability of the models to identify active compounds was evaluated by using receiver operator characteristic (ROC) curves based on a ranked list of the ligands and decoys. The ligand enrichment was quantified using the logAUC metric [33], which is calculated from the area under a semi-logarithmic version of the ROC curve and adjusted by subtracting the area corresponding to random enrichment. The logAUC metric favors early ligand enrichment, which is important in virtual screening, and a positive value indicates that the model performs better than random selection. The ROC-based enrichment factors at 1% (EF1) were also calculated (Fig I in S1 Appendix), but as these led to similar conclusions, the presented analysis is based on the LogAUC values.

**Fig 10:**
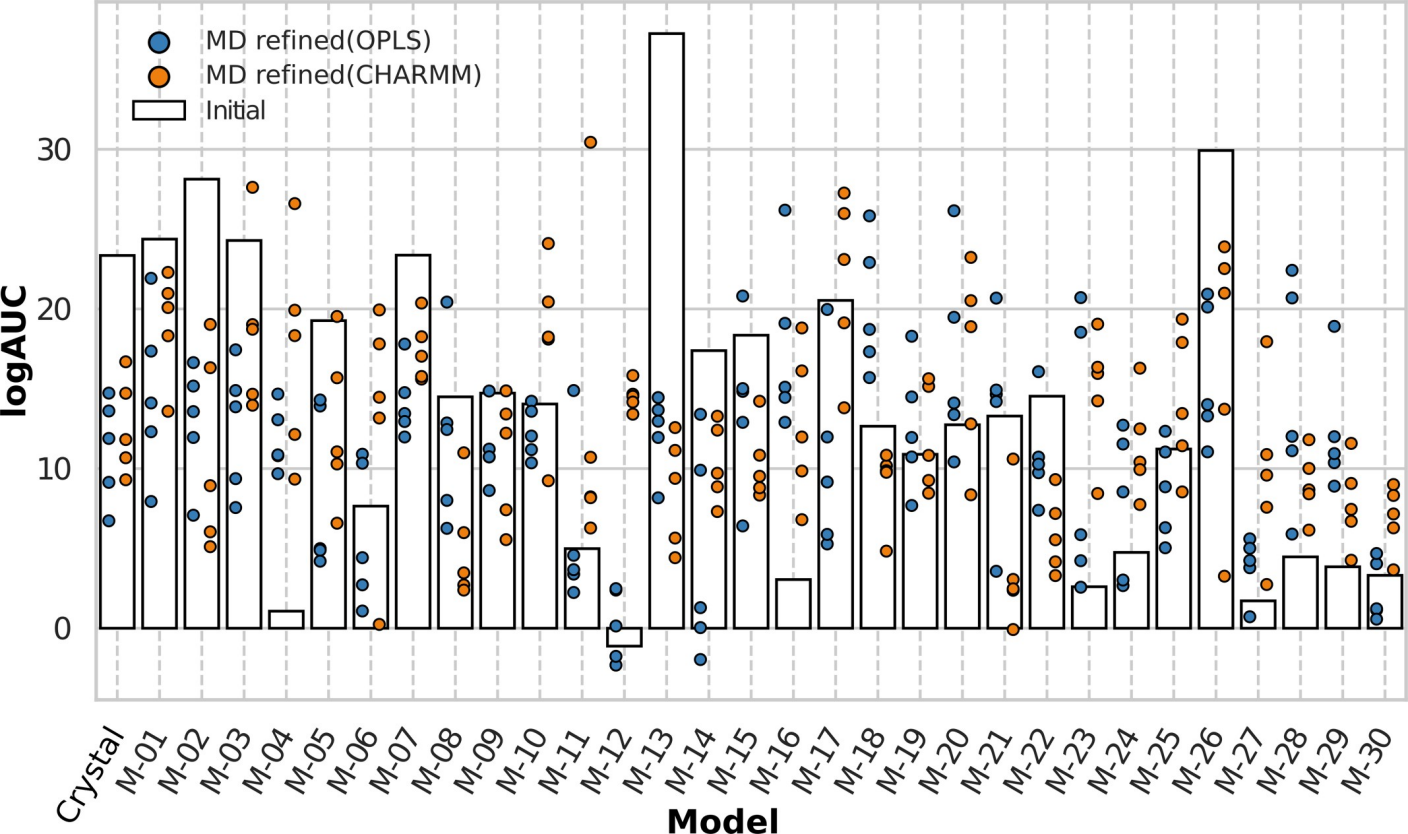
Virtual screening performance of the initial and MD refined structures. Ligand enrichment was assessed using logAUC values. The results for the crystal structure and initial models are shown as bars. The logAUC values for the five MD refined models and crystal structures are shown as blue and orange circles for the OPLS and CHARMM protocols, respectively.

Overall, MD refinement did not improve virtual screening performance. The median change in logAUC was –1.9 and +0.5 after MD refinement with the OPLS and CHARMM protocols, respectively. Hence, around half of the models showed improved enrichment. Prior to the MD refinement, there was a trend towards better ligand enrichment for the GPCR Dock models with good predictions of the ligand binding mode, but there were large variations in enrichment within each group (Fig 10). The 10 GPCR Dock models within the good range of RMSD_LIG_ values had a median logAUC of 17%. The corresponding median logAUC values were 13% and 4% for models in the medium and bad RMSD_LIG_ range, respectively. Seven models had better ligand enrichment than the crystal structure, which had a logAUC of 23% and the binding mode of eticlopride was well predicted in four of these. However, it should be noted that there were also models with poor virtual screening performance among the models with good prediction accuracy for the ligand, *e*.*g*. M-04. This was not unexpected as ligand enrichment will also depend on the accuracy of loop prediction and the binding site. MD refinement had a strong impact on the ligand enrichment. After MD refinement, the GPCR Dock models with good initial RMSD_LIG_ values generally retained good ligand enrichment values for at least one centroid. For these models, the median of the best logAUC values for each MD refinement were 16% and 20% for the OPLS and CHARMM protocols, respectively. Interestingly, the overall trend was that the GPCR Dock models that displayed good enrichment performed slightly worse after MD refinement whereas models that initially had poor logAUC values improved. For several good GPCR Dock models that enriched ligands well, the loss of enrichment could be explained by a less accurate eticlopride binding mode after MD refinement (e.g. M-01, M-02, and M-05). Conversely, the accuracy of the binding mode of eticlopride improved for M-04 and the ligand enrichment was also enhanced in this case. For most of the GPCR Dock models with medium to bad RMSD_LIG_ values (RMSD_LIG_ > 2.5 Å), the enrichments improved for at least one of the MD refined models. For the 17 models with medium accuracy of the ligand binding mode, the median best logAUC improved by 5 and 3 logAUC units for the OPLS (logAUC = 18%) and CHARMM (logAUC = 16%), respectively.

Two GPCR Dock models (M-04 and M-12) were analyzed in more detail. Both models had very low logAUC values that indicated that ligand enrichment was only slightly better or even worse than random selection. After MD refinement with the CHARMM protocol, the ligand enrichment increased for all the centroids to values between 9 and 27%. Inspection of these models showed that there were both improvements of the predicted eticlopride binding mode and receptor structure in these models (Fig 2C). For M-04, the initial ligand binding mode (RMSD_LIG_ = 1.8 Å) formed the key salt bridge with Asp110, but the aromatic ring was slightly displaced due to modeling errors in EL2 and the top parts of TM helix 5 and 6. After MD refinement, the helices reoriented to a conformation that was more similar to the crystal structure. This movement was also accompanied by a refinement of the part of EL2 that is closest to the binding site, and the ligand could access the hydrophobic region formed between the loop and the two helices with a maintained salt bridge. As a consequence, the ligand reached poses that were more similar to the crystal structure with RMSD_LIG_ values ranging from 1.0 Å and 1.7 Å. These changes in the binding site increased the logAUC to values between 9 and 27%. In the M-12 model, the ligand lacked the salt bridge interaction with Asp110. After the MD refinement, the salt bridge was formed and the ligand binding mode improved in four models. In one case, the RMSD_LIG_ decreased from 3.1 Å to 1.1 Å and the logAUC value increased from –1.4 to 16%.

## Discussion

The goal of this study was to assess if models of GPCRs could be refined by MD simulations to improve predictions of receptor-ligand complexes. Two different MD simulation protocols were explored and comparison of refined models to the crystal structure led to four main results. First, it is unlikely that MD refinement improves the TM region of GPCR models, which drifted further away from the crystal structure during the simulation. Second, the best MD refinement protocol was able to refine a majority of the ligand binding modes and large improvements were achieved in a few cases. Third, application of restraints to maintain the TM region close to the initial model improved predictions of both the loop region and the binding mode of the ligand. Finally, the virtual screening performance of the receptor models could be improved by MD refinement.

There were clear differences between the performance of the two MD simulation protocols. Compared to OPLS, the CHARMM protocol resulted in higher accuracy in the TM region. Whereas the CHARMM simulations refined almost 40% of the models in the TM region, OPLS improved only one model (3%). The CHARMM protocol consistently produced better ligand poses. Larger improvements of the ligand binding mode were obtained both in CHARMM simulations of the GPCR Dock models and complexes obtained by docking to the crystal structure. In addition, the CHARMM refined structures also showed better virtual screening performance. It is difficult to draw definitive conclusions regarding the role of the protein force field as there were several differences between the protocols, including details regarding system preparation, ligand and membrane parameterization, equilibration, and MD simulation software. However, some of the observed differences between the two simulation protocols were likely due to the force fields. For example, CHARMM protocol resulted in better protein structure quality scores than OPLS, indicating that the CHARMM36m force field describes interactions more accurately. The fact that the OPLS-AA/L force field is older than CHARMM36m suggests that improvements of parameters in the more modern protein force fields will lead to better refinement results [34], which is encouraging.

MD simulations have been used successfully to fold proteins to native-like structures, but this approach is limited to short amino acid sequences [35]. MD refinement of homology models based on good templates, which will have a relatively high overall accuracy in secondary and tertiary structure, may appear to be more feasible. However, we found that the TM region of our MD refined GPCR structures drifted further away from the crystal structure with both simulation protocols. This observation is in agreement with a benchmark on globular proteins by Raval *et al*., which showed that MD refinement of models from the CASP assessment also drifted further away from the native structure [36]. In the loop region, we found that both protocols could generate at least one improved structure (out of five) for about half of the models. One reason to why the results for EL2 appear to be slightly better than the TM region could be that the loop regions were poorly predicted in GPCR Dock 2010 [14], which leaves more room for improvement. The overall lack of structural refinement for the receptor can be explained by a combination of limited conformational sampling, force field accuracy, and uncertainties related to experimental structure determination. In our simulations of the D_3_R, the GPCR Dock models were clearly less stable than the crystal structure. Although the TM region was well predicted in many cases, the presence of several small modeling errors (*e*.*g*. strained backbone or clashing side chains) rapidly pushed the receptor structure away from the initial conformation. The path to the more native-like structures of the TM region sampled in the simulation of the D_3_R crystal structure may involve crossing high energy barriers, which is not within reach of the short time-scales possible with all atom MD simulations. However, it is also possible that the experimental structure is not a global energy minimum of the force field model. For example, Raval *et al*. showed that experimental structures also drifted further away from the initial conformation in microsecond simulations [36]. Such observations may be due to differences in conditions compared to the crystallographic experiment to some extent (*e*.*g*. the difference in treatment of IL3 in the simulations compared to the crystal structure in our case), but also suggests that improved force fields are needed. The current limitations of force fields and sampling explain why protocols that use relatively short simulations with restraints to limit large conformational changes have been most successful in refining protein models in CASP [10,37]. This approach may be particularly suitable for class A GPCRs as the structure in the TM region is relatively conserved. Encouragingly, we found that the simple approach of applying weak restraints to the C_α_ atoms of the TM helices improved agreement with the D_3_R crystal structure compared to unrestrained simulations. Encouragingly, the percentage of models refined with the OPLS protocol increased from 3% to 43% with improvements of up to –0.14 Å. The best refinements are similar to the results obtained by Dutagaci *et al*. for a set of eight membrane proteins [38] using a protocol developed for soluble proteins that performed well in the CASP assessment [39,40]. In their study, two out of three GPCR models were refined in the helical regions with RMSD improvements of –0.06 and –0.18 Å. Another encouraging result from our restrained simulations was the enhanced predictions for the (unrestrained) loop region and ligand. By restraining parts of the receptor that are likely to be well modelled, sampling is focused on other regions that need further refinement. The optimal length of the simulations likely depends on several factors (*e.g.* the use of restraints and expected accuracy of the initial model), but several studies suggest that the use of multiple replicas with lengths of around 100 to 200 ns is suitable for model refinement and yields statistical robustness [41–43]. The recent development of more tailored refinement protocols that apply restraints based on the anticipated local accuracy of the model [44] or contacts that are conserved [45,46] could further improve GPCR models.

Accurate modeling of protein-ligand complexes is essential in structure-based drug design. As structure determination for GPCRs is still very challenging, reliable methods to predict ligand binding modes would be very valuable. A majority of the 30 models studied in this work, which were generated by 25 participants of GPCR Dock 2010 [14], were obtained by molecular docking of the ligand to the binding site. We investigated if an MD refinement would have resulted in a more accurate prediction of the crystal structure. Our results show that if five complexes are generated with the best MD refinement protocol, 73% of these sets will contain a more accurate representation of the complex and a large fraction of these (>40%) will represent large improvements of >1 Å. If a single complex is generated with the best MD refinement protocol, 57% of the models will be improved. In several cases, large conformational changes in the binding site were necessary to reach the improved pose, which would not have been possible to achieve by standard molecular docking due to the limited representation of induced fit. Considering that our approach is largely automated, the effort to generate and inspect additional complexes as part of lead optimization process is relatively small. Prediction accuracy could likely be further improved by using more advanced methods for selecting representative snapshots from the MD simulations [47,48] and incorporation of target-specific knowledge regarding ligand-residue contacts, mutagenesis data, and structure-activity relationships for known ligands [49–52].

Three important observations were made from a series of simulations of the D_3_R crystal structure with ligand binding modes of varying accuracy. First, difficulties to refine the ligand binding mode will persist even if an accurate receptor model is used in the MD simulations. As in the case of the GPCR Dock models, we obtained improved predictions of ligand binding modes for about half of the complexes. The only notable difference was the large improvements obtained for a few models with an initial RMSD_LIG_ > 5 Å, which suggest that an accurate receptor model may facilitate refinement of poorly predicted ligand binding modes. Second, the potential to refine the structure of a receptor-ligand complex depends on the nature of conformational changes necessary and is weakly correlated with how close the initial pose is to the experimental structure. Overall, it appeared more difficult to refine ligand poses that initially are close to the crystal structure. In agreement with these results, Feig *et al*. reported a similar behavior of accurately modelled regions of proteins, which showed a tendency to deviate after MD compared to amino acids in less accurate regions [39]. Third, that a ligand remains stable in an MD simulation over relatively long time-scales does not support that the predicted binding mode is accurate. As demonstrated by long-time scale simulations by Dror *et al*., ligands can reside in metastable states for microseconds [53, 54]. In our simulations, binding modes that were both close and distant to the experimentally observed pose could remain stable in the simulations over 0.5 μs. Given that conformational sampling is a limiting factor for refinement of binding modes, it would be interesting to further explore enhanced sampling approaches in prediction of GPCR-ligand complexes [55–57].

An important application of protein structures is virtual screening of chemical libraries to identify novel ligands [58]. Molecular docking screening using GPCR crystal structures has been remarkably successful and led to the discovery of leads for several therapeutically important targets [59–61]. Improved refinement of homology models could make it possible to extend the use of virtual screening to the many GPCRs for which there are no available experimental structures. As demonstrated by Jaiteh *et al*., an improved binding site model can substantially improve enrichment of ligands in a chemical library [49] and there are several successful virtual screens based on GPCR homology models [62–65]. Previous studies have also demonstrated that refinement of GPCR models guided by docking to multiple binding site conformations can improve virtual screening performance [51,66–69]. In the same vein, we investigated the virtual screening performance of the GPCR Dock models before and after MD refinement. Interestingly, models displaying improved ligand enrichment were obtained after MD refinement in a majority of the cases. Encouragingly, the largest improvements were obtained for models that initially had poor enrichment. However, we also noted that performance was reduced for some of the good GPCR Dock models with high enrichment, which was likely due to that the MD refinement led to less accurate ligand poses in these cases. Pragmatically, this is not a problem if a set of known ligands are available for the target GPCR. In such cases, virtual screening performance of models can be benchmarked and the best performing binding site structure is identified. If a set of ligands is not available to assess the models, selection of a structure from MD refinement is actually equally likely to result in better or worse ligand enrichment. It is also clear from previous studies that, although MD simulations can generate GPCR models with good virtual screening performance [70,71], using multiple GPCR homology models may be more cost-efficient and yields comparable performance [49]. As homology modeling and MD simulations tend to explore different conformational ensembles, the best strategy may be to combine the approaches.

During the last decade, the number of experimentally determined GPCR structures has increased rapidly. MD simulations based on these structures have proven very valuable in studies of both receptor function and drug binding. Long-timescale simulations have been used to understand the GPCR activation mechanism, the role of water networks for receptor function, and the ligand binding process [72–75]. Furthermore, MD simulations in combination with free energy calculations have also been shown to be useful in lead optimization, evaluation of ligand binding modes, and assessment of ligand selectivity [76–78]. In all of these studies, access to atomic resolution structures of GPCRs was essential. Although structure prediction methods have improved during the last 10 years [79,80], homology modeling accuracy remains limited by that the sequence identity to available templates is <40% in most cases [80]. We assessed if MD simulations can be used to refine models of GPCR-ligand complexes, which primarily had been obtained using homology modelling combined with molecular docking calculations. Whereas the overall receptor structure was difficult to refine, we show that more accurate ligand binding modes can be obtained and that MD snapshots can show improved virtual screening performance. Our results also suggest that enhanced force fields and restraints in the MD simulations have a positive effect on model accuracy, which can guide further development of structure refinement methods.

## Methods

### OPLS protocol

The crystal structure of the D_3_R (PDB code: 3PBL, chain A) in complex with the antagonist eticlopride (ETQ) was first inserted into a hexagonally shaped bilayer with 291 POPC lipids and subsequently solvated with 16,000 water molecules to a ratio of ~50 water molecules per POPC lipid. Prior to this step the missing side chains of residues Gln144, Ser145, and Thr357 were added using MODELLER [81]. The initial preparation was performed with PyMemDyn, which is part of the GPCR modelling pipeline of the GPCR-ModSim server[45]. The lipids were modelled with the Berger parameters [82], and subsequently treated with the half-ε, double pair-list method [83] to make the scaling factors compatible with the OPLS-AA/L[17] force field parameters. A sodium ion together with 10 crystal water molecules were aligned into the protein from a β_1_ adrenergic receptor structure (PDB code: 4BVN) [84]. The models from GPCR Dock 2010 were downloaded from the assessment webpage [14,23] and converted to PDB format. GPCR Dock Models were primarily selected based on their ligand score. First, the highest ranked model from each participant was considered. If a model failed in the simulation preparation, it was discarded and a model with a similar ligand RMSD from the assessment was selected. The 30 selected models are summarized in Table F in S1 Appendix. The models were truncated to match the sequence of the crystal structure and inserted into the same POPC bilayer by alignment of the TM heavy atoms to the crystal structure. Water molecules clashing with the model structures were removed and 0.15 M NaCl was added. The protein topologies were prepared for the OPLS-AA/L force field with the pdb2gmx tool in GROMACS 5.1.2 [18,85,86]. The ligand parameters (OPLS-AA 2005) were prepared with Schrödinger Macromodel 11.3 (http://www.schrodinger.com) and water molecules were modelled with the SPC [87] water model.

The models and the crystal structure reference were minimized with a steepest descent algorithm with an energy step size of 0.01 and energy tolerance 1000 kJ mol^-1^ nm^-1^ for 5000 steps or until converged to machine precision. During the equilibration phase, the minimized models were initially position restrained on all heavy atoms (including the ligand) with a force constant of 1000 kJ mol^-1^ nm^-2^. The restraints were then released in steps of 200 kJ mol^-1^ nm^-2^ every 5 ns down to k = 200 kJ mol^-1^ nm^-2^, followed by 5 ns C_α_-restraints (k = 200 kJ mol^-1^ nm^-2^) and 5 ns unrestrained simulations. During equilibration, the v-rescale (Bussi)[88] thermostat and Berendsen barostat[89] were employed with coupling constants of 0.5 and 2.0 ps, respectively. The reference temperature and pressure were 310 K and 1.0 bar, respectively. The pressure was coupled to the barostat in a semi-isotropic manner (separately for xy- and z-dimensions). After equilibration, three 100 ns simulations equilibrated with different starting velocities were performed using the Parrinello-Rahman barostat[90] with a coupling constant of 12 ps. The compressibility constant was set to 4.5x10^-5^ bar^-1^. The temperature coupling was divided to handle water and ions, protein and ligand, and POPC, separately. The bonds in water and solutes (lipids and protein) were constrained using SETTLE[91] and LINCS[92,93] algorithms, respectively. Outside the real space cut-off of 1.2 nm, electrostatic interactions were handled with the Particle Mesh Ewald[94] algorithm with cubic interpolation and a fourier spacing of 0.15 nm. All van der Waals interactions were truncated at a cut-off distance of 1.2 nm. The simulations were performed in GROMACS 5.1.2[18,86] with a leap-frog time step of 2 fs. A set of simulations with restraints (k = 80 kJ mol^-1^ nm^-2^) on the Cα atoms in the TM region (residues 32–57, 62–92, 100–132, 146–168, 186–214, 322–354 and 361–385) was also carried out. In this case, the last equilibration step was a simulation of 10 ns with C_α_ restraints (k = 80 kJ mol^-1^ nm^-2^). These simulations were performed with GROMACS v2020.2[95].

### CHARMM protocol

The second protocol was based on the GPCR simulation pipeline developed by the GPCRmd database [96]. The OPLS topologies were translated into CHARMM topologies by using an in-house script using the HTMD 1.9.9 software package [97]. The models were aligned to the OPM [98] database crystal structure of D_3_R in complex with eticlopride (PDB code: 3PBL) using STAMP 4.4 [16,99,100]. The receptor was embedded into a POPC bilayer and solvated ensuring a 20 Å distance between protein periodic distances considering also receptor diffusional rotation. Systems were solvated with TIP3P water molecules and the ionic strength of the solution was kept at 0.15 M with NaCl ions. Parameters for the simulation were obtained from the CHARMM36m force field [19,101–107]. Parameters for the ligand were assigned from the CGenFF 3.0.1 force field automatically by the ParamChem tool 1.0.0 [20,108–110]. An initial 5000 step minimization was followed by an equilibration of 40 ns at constant pressure (NPT, 1.01325 bar) using the Berendsen barostat with a pressure relaxation time of 800 fs and a compressibility factor of 4.57x10^-5^ bar^-1^. In a first step, harmonic restraints of 1.0 kcal mol^-1^ Å^-2^ were applied to the protein backbone, the sodium ion in the TM region, waters derived from crystal structures, and ligand heavy-atoms during 20 ns. The restraints were then gradually removed during 10 ns (-0.095 kcal mol^-1^ Å^-2^ ns^-1^). Finally, restraints where removed in a final 10 ns equilibration step. After the NPT steps, a production simulation at conditions of constant volume (NVT) was performed in three replicates for each of the models and the crystal structure (100 ns). These simulations were performed in ACEMD [21]. The equilibration and production steps used a time step of 2 and 4 fs, respectively. Selection of these time steps was possible due to the hydrogen mass repartitioning scheme being employed in ACEMD [111]. The non-bonded interaction cut-off was set at 9 Å. A smooth switching function for the cut-off was applied, starting at 7.5 Å. The size of the cell was set to prevent non-bonded interactions between the protein and its periodic boundary image. Long-distance electrostatic forces were calculated using the Particle Mesh Ewald [112] algorithm using a grid spacing of 1 Å. The bond lengths of hydrogen atoms were kept constrained using the RATTLE [113] algorithm. Simulations were carried out at a temperature of 310 K using a Langevin thermostat with a damping constant γ of 1 ps^-1^ in NPT simulations and 0.1 ps^-1^ in NVT.

### Docking of eticlopride to the D_3_R crystal structure

The D_3_R crystal structure was used to perform redocking calculations of eticlopride. A conformational search of eticlopride was performed using the obconformer tool (the Open Babel Package, version 2.4.1)[114] to generate geometrically optimized conformers (NSteps and GeomSteps were set to 250). The best conformer was further refined through conjugate gradient and steepest descent algorithms by using the obminimize tool (the Open Babel Package, version 2.4.1) with default settings. AutoDock [115] (release 4.2.6) was used to perform the docking calculations because of its ability to generate and cluster multiple ligand binding modes. Docking input files were prepared through the AutoDockTools (ADT) package[115] using a grid of 40x80x110 points with a spacing of 0.375 Å. AutoGrid4 was used to generate grid maps. The Lamarckian genetic algorithm (LGA) was employed with a population size of 300 individuals, a maximum number of 2,500,000 energy evaluations, a maximum number of 27,000 generations, and 100 runs. Predicted poses obtained from AutoDock were clustered based on their Hungarian RMSD value from the experimental pose of eticlopride. Ten diverse poses of eticlopride were selected based on visual inspection. These complexes were subsequently prepared and simulated according to the OPLS and CHARMM protocols as described above. Three replicates of 500 ns were generated for each complex.

### Analysis of MD simulations

#### Clustering of the trajectories

The three replicate trajectories from each production MD run were concatenated, treated for periodic boundaries, and aligned (rotation+translation) to the crystal structure by minimizing the TM RMSD using the GROMACS tool trjconv. The resulting trajectories contained ~1500 snapshots, corresponding to one snapshot every 200 ps. The snapshots were clustered based on ligand heavy atom RMSD by feeding an RMSD distance matrix to the affinity propagation routines from SciKit Learn[116] through the Encore clustering module of the MDAnalysis (v.0.17.0) python package [117–119]. Affinity propagation clustering was selected because this method was able to consistently produce at least five diverse cluster centers after tuning the preference input parameter (preference = –8.0). The five largest clusters from each model were selected for further analysis.

#### RMSD and RMSF calculations

The centroids from the five largest clusters, the initial structure for each of the simulated models, and the simulated crystal structure were analysed with respect to ligand heavy atom RMSD (RMSD_LIG_) as well as TM (RMSD_TM_, residues 32–57, 62–92, 100–132, 146–168, 186–214, 322–354 and 361–385) and EL2 (RMSD_EL2_, residues 171–185) backbone RMSD with the crystal structure as reference. RMSD and RMSF values were calculated with the MDAnalysis (v.0.17.0) python package[117,118]. In addition, side chain RMSDs (RMSD_SC_) were calculated for 15 residues within 4 Å of the ligand in the crystal structure (Phe106, Asp110, Val111, Cys114, Ile183, Val189, Ser192, Trp342, Phe345, Phe346, His349, Val350, Tyr365, Thr369 and Tyr373). The RMSD_SC_ was calculated with sPyRMSD [120] to obtain symmetry corrected values.

#### MolProbity and n-DOPE calculations

All structures were first aligned to the initial crystal structure prepared using the OPLS protocol. Prior to the calculation, the membrane, solvent, salt, hydrogens, ligand, and terminus caps were removed. The MolProbity scores were calculated using the MolProbity[28] module in Phenix[121] with default settings. The aligned structures were also used to calculate the normalized DOPE [29,30] scores (n-DOPE) using MODELLER v9.19 [81] with default settings.

#### Molecular docking and ligand enrichment

Each of the initial models and their corresponding five cluster centroids were aligned to the OPLS prepared crystal structure and prepared for docking with DOCK v3.7 [32] using PyMOL v2.3.0 [122]. Hydrogen positions from the MD preparations and simulations were maintained. Docking grids were generated using the program blastermaster with default parameters. Matching and low dielectric spheres were generated with SPHGEN [123]. The binding energy was calculated as the sum of electrostatic and van der Waals interaction terms, corrected for ligand desolvation. These energy terms were calculated from precalculated grids with a default box size. The electrostatic grid was calculated using Qnifft [124]. The van der Waals grid was calculated with Chemgrid [125] using a cut off of 10 Å and with a grid spacing of 0.2 Å. The ligand desolvation grid was calculated with SOLVMAP [33].

The DUD-E dataset for D_3_R [31,32], which contained 475 ligands and 33,827 decoys, was downloaded from http://files.docking.org [126]. This library was docked with DOCK 3.7 [32] to the receptor models. The ligands were matched with the adaptive sampling routine with an initial distance tolerance of 0.05 Å and a step size of 0.05 Å until either of the maximum distance tolerance of 0.5 Å or 5000 conformations was reached. A bump limit of 50 kcal/mol was used for both the flexible and rigid parts of the ligands. The best scoring conformation was minimized for a maximum of 500 steps or until converged to 0.1 kcal/mol with translational and rotational step sizes of 0.2 Å and 5.0 degrees, respectively. The semi-log ROC curves and corresponding adjusted logAUC[33] and EF1 [31] values were calculated with the program enrich.py included with the DOCK 3.7 package. The values for the initial models were taken as the average logAUC between the CHARMM and OPLS prepared structures, which resulted in slightly different values due to differences in the OPLS and CHARMM protocols.

## Supporting information

S1 Appendix Supporting information for MD simulations and molecular docking calculations. **Table A.** Protocols used in simulations of the D_3_R models and crystal structure. **Table B.** Percentage of improved models after MD refinement based on analysis of side chains in the binding site. **Table C.** Accuracy of the binding site side chains based on the difference in RMSD for the refined models compared to the initial model (ΔRMSD = RMSD_MD refined_−RMSD_Initial model_). **Table D.** Percentage of improved models after MD refinement depending on the number of included cluster centroids (1–5). The analysis is based on the RMSD of the ligand (RMSD_LIG_). **Table E.** Accuracy of the TM region, EL2, and ligand (LIG) after MD refinement with restraints based on the difference in RMSD compared to the initial model (ΔRMSD = RMSD_MD refined_−RMSD_Initial model_). **Table F.** D_3_R models from the GPCR Dock 2010 that were used for simulations with the OPLS and CHARMM protocols. **Fig A.** Comparison of RMSD values calculated for the TM, EL2, and ligand (LIG) in this work using rotational/translational least squares fit of the TM region (RMSD_RT_) and the GPCR Dock 2010 assessment (RMSD_A_). The model M-30 (2364_5_) was treated as an outlier and is not included in the comparison. **Fig B.** Fluctuations in the TM region of D_3_R crystal structure and models. Average RMSF values for TM region from the simulations performed with the OPLS (blue) and CHARMM (orange) protocols. **Fig C.** RMSD_TM_ calculated for each model and simulation protocol with the initial structures as reference. The three replicate simulations are averaged for OPLS (blue) and CHARMM (orange) at every snapshot in time. The standard error at 95% confidence interval is shown in paler colors. **Fig D.** Effect of MD refinement on protein quality scores. (A) MolProbity and (B) n-DOPE scores for the initial (bars) and MD refined (circles) structures using the OPLS (blue) and CHARMM (orange) protocols. **Fig E.** RMSD_LIG_ calculated for each model and simulation protocol with the initial structures as reference. The three replicate simulations are averaged for OPLS (blue) and CHARMM (orange) at every snapshot in time. The standard error at 95% confidence interval is shown in paler color. **Fig F.** Ligand poses from the simulations initiated from the crystal structure of D_3_R. The receptor is shown as cartoons and the ligand in sticks. The best MD refined models and the crystal structure are colored green and grey, respectively. **Fig G.** Assessment of the effect of simulation length on RMSD_LIG_. (A) The change in RMSD (ΔRMSD_LIG_) averaged in blocks of 10 ns over all three replicates of all MD refinement simulations. The distribution of the binding modes with the best RMSD_LIG_ values for the (B) OPLS and (C) CHARMM protocols based on the centroids representing the five largest clusters for each model. **Fig H.** Best ΔRMSD_TM_ (difference in RMSD_TM_ between the best MD refined and initial structure) from different simulation protocols. Data from unrestrained CHARMM (yellow), OPLS (black), and restrained OPLS (red) simulations are shown. **Fig I.** Virtual screening performance of the initial and MD refined structures. EF1 results for the crystal structure and initial models are shown as bars. The EF1 values for the five MD refined models and crystal structures are shown as blue and orange circles for the OPLS and CHARMM protocols, respectively.
(PDF)
