# Supplementary material for: Can molecular dynamics simulations improve the structural accuracy and virtual screening performance of GPCR models?
Source: PLoS Comput Biol. 2021 May 13;17(5):e1008936. doi: 10.1371/journal.pcbi.1008936 (PMC8186765; doi:10.1371/journal.pcbi.1008936)
Supplement: S1 Appendix — Table A. Protocols used in simulations of the D3R models and crystal structure. Table B. Percentage of improved models after MD refinement based on analysis of side chains in the binding site. Table C. Accuracy of the binding site side chains based on the difference in RMSD for the refined models compared to the initial model (ΔRMSD = RMSDMD refined−RMSDInitial model). Table D. Percentage of improved models after MD refinement depending on the number of included cluster centroids (1–5). The analysis is based on the RMSD of the ligand (RMSDLIG). Table E. Accuracy of the TM region, EL2, and ligand (LIG) after MD refinement with restraints based on the difference in RMSD compared to the initial model (ΔRMSD = RMSDMD refined−RMSDInitial model). Table F. D3R models from the GPCR Dock 2010 that were used for simulations with the OPLS and CHARMM protocols. Fig A. Comparison of RMSD values calculated for the TM, EL2, and ligand (LIG) in this work using rotational/translational least squares fit of the TM region (RMSDRT) and the GPCR Dock 2010 assessment (RMSDA). The model M-30 (23645) was treated as an outlier and is not included in the comparison. Fig B. Fluctuations in the TM region of D3R crystal structure and models. Average RMSF values for TM region from the simulations performed with the OPLS (blue) and CHARMM (orange) protocols. Fig C. RMSDTM calculated for each model and simulation protocol with the initial structures as reference. The three replicate simulations are averaged for OPLS (blue) and CHARMM (orange) at every snapshot in time. The standard error at 95% confidence interval is shown in paler colors. Fig D. Effect of MD refinement on protein quality scores. (A) MolProbity and (B) n-DOPE scores for the initial (bars) and MD refined (circles) structures using the OPLS (blue) and CHARMM (orange) protocols. Fig E. RMSDLIG calculated for each model and simulation protocol with the initial structures as reference. The three replicate simulations are averaged for O [file pcbi.1008936.s001.pdf]

S1 Appendix:

Can molecular dynamics simulations improve the structural accuracy and virtual screening performance of GPCR models?

Jon Kapla[1], Ismael Rodriguez Espigares[2], Flavio Ballante[1], Jana Selent[2], Jens Carlsson[1,*]

[1] Science for Life Laboratory, Department of Cell and Molecular Biology, Uppsala University, Uppsala, Sweden.
[2] Research Programme on Biomedical Informatics (GRIB), Department of Experimental and Health Sciences of Pompeu Fabra University (UPF), Hospital del Mar Medical Research Institute (IMIM), Barcelona, Spain.

Table of contents

**Table A.**	Simulation protocols	**3**
**Table B.**	Percentage of improved models after refinement based on binding site side chains	**4**
**Table C.**	Accuracy of the binding site side chains after MD refinement	**5**
**Table D.**	Percentage of improved models depending on the number of included cluster centroids	**6**
**Table E.**	Median RMSD changes after restrained MD refinement	**7**
**Table F.**	Selected models	**8**
**Fig A.**	Alignment to crystal structure and RMSD values	**9**
**Fig B.**	Model RMSF in the TM region	**10**
**Fig C.**	$RMSD_{TM}$ averaged over replicates	**11**
**Fig D.**	MolProbity and n-DOPE scores	**12**
**Fig E.**	$RMSD_{LIG}$ averaged over replicates	**13**
**Fig F.**	Ligand poses from the crystal structure simulations	**14**
**Fig G.**	Assessment of simulation time	**15**
**Fig H.**	Per model $\Delta RMSD_{TM}$ from different simulation protocols	**16**
**Fig I.**	Virtual screening performance based on EF1	**17**

References **18**

Table A. Protocols used in simulations of the D_3R models and crystal structure.

	**OPLS**	**CHARMM**
Simulation software	Gromacs[1-3]	ACEMD[4]
**Force field parameters**		
Protein/Salt	OPLS-AA[5]	CHARMM36m[6]
Lipid	Berger[7]	CHARMM36[8]
Ligand	Schrödinger/OPLS2005[9]	CGenFF[10-12]
Water	SPC [13]	TIP3P[14]
**Production simulation parameters[a]**		
Ensemble	NPT	NVT
Barostat	Parrinello-Rahman[15]	-
Thermostat	v-rescale (Bussi)[16]	Langevin[4]

[a] Details regarding system preparation are described in the methods section.

Table B. Percentage of improved models after MD refinement based on analysis of side chains in the binding site.

RMSD$_{SC}$ selection[a]	OPLS / %[b]	CHARMM / %[b]
**All**	19	38
**Phe106**	34	52
**Asp110**	34	43
**Val111**	31	27
**Cys114**	52	49
**Ile183**	34	43
**Val189**	32	29
**Ser192**	35	59
**Trp342**	9	30
**Phe345**	19	32
**Phe346**	20	33
**His349**	39	59
**Val350**	41	37
**Tyr365**	31	51
**Thr369**	43	52
**Tyr373**	43	39

[a] Residues within 4 Å of the ligand in the D_3R crystal structure (15 residues).
[b] Calculated based on the centroids representing the five largest clusters from each MD refinement. Models M-08 and M-09 were excluded from the calculations due to errors in the atom naming in the initial model.

Table C. Accuracy of the binding site side chains based on the difference in RMSD for the refined models compared to the initial model ($\Delta RMSD = RMSD_{MD\ refined} - RMSD_{Initial\ model}$).

**OPLS**			
**Model quality**[a]		**ΔRMSD/Å**[b]	**$\Delta RMSD_{Best}$/Å**[c]
**All**		0.78	0.34
**Good**	0.0 – 2.5 Å	0.72	–0.10
**Medium**	2.5 – 5.0 Å	0.79	0.42
**Bad**	> 5.0 Å	1.0	0.34
**CHARMM**			
**Model quality**[a]		**ΔRMSD/Å**[b]	**$\Delta RMSD_{Best}$/Å**[c]
**All**		0.13	–0.18
**Good**	0.0 – 2.5 Å	–0.28	–0.74
**Medium**	2.5 – 5.0 Å	0.35	0.05
**Bad**	> 5.0 Å	0.68	0.33

[a] Based on the $RMSD_{LIG}$ of the GPCR Dock models. Models M-08 and M-09 were excluded from the calculations due to errors in the atom naming in the initial model.
[b] Calculated based on the median of the centroids representing the five largest clusters from each MD refinement, *i.e.* the third best result of each MD refinement is used to calculate the $\Delta RMSD_{SC}$.
[c] Calculated based on the best $RMSD_{SC}$ value obtained from the centroids representing the five largest clusters from each MD refinement.

Table D. Percentage of improved models after MD refinement depending on the number of included cluster centroids (1-5). The analysis is based on the RMSD of the ligand ($RMSD_{LIG}$).

Clusters[a]	OPLS[b] / %	CHARMM[b] / %
**1**	27	57
**1+2**	37	60
**1+2+3**	43	70
**1+2+3+4**	50	73
**1+2+3+4+5**	50	73

[a] The centroids representing the five largest clusters from each MD refinement were ranked by cluster size. The first cluster (1) is the largest and the fifth (5) cluster is the smallest.
[b] Calculated based on the best $RMSD_{LIG}$ value from the included clusters from the MD refinement.

Table E. Accuracy of the TM region, EL2, and ligand (LIG) after MD refinement with restraints based on the difference in RMSD compared to the initial model ($\Delta RMSD = RMSD_{MD\ refined} - RMSD_{Initial\ model}$).

**OPLS (restrained) protocol**[a] **Model quality**[b]		**Selection**	**ΔRMSD/Å**[c]	**$\Delta RMSD_{Best}$/Å**[d]	**$\Delta RMSD_{Min}$/Å**[e]
**All**		**LIG**	0.38	–0.11	–0.63
		**TM**	0.14	0.05	-0.11
		**EL2**	0.06	–0.61	–1.3
**Good**	0.0 – 2.5 Å	**LIG**	1.0	0.23	–0.37
	0.0 – 1.5 Å	**TM**	0.19	0.08	–0.09
	0.0 – 4.0 Å	**EL2**	0.46	–0.03	–1.0
**Medium**	2.5 – 5.0 Å	**LIG**	0.22	–0.23	–0.8
	1.5 – 2.5 Å	**TM**	0.11	0.00	–0.14
	4.0 – 5.0 Å	**EL2**	0.12	-0.65	–1.4
**Bad**	> 5.0 Å	**LIG**	–0.77	–1.3	–3.2
	> 2.5 Å	**TM**	0.04	–0.01	–0.17
	> 5.0 Å	**EL2**	–0.28	–1.4	–2.2

[a] The OPLS protocol with restraints in the TM region (C_α atoms).
[b] Based on the RMSD values of the GPCR Dock models.
[c] Calculated based on the median of the centroids representing the five largest clusters from each MD refinement, *i.e.* the third best result of each MD refinement is used to calculate the ΔRMSD.
[d] Calculated based on the best RMSD value obtained from the centroids representing the five largest clusters from each MD refinement.
[e] Calculated based on the minimum RMSD identified in all 1500 snapshots generated for each model.

Table F. D_3R models from the GPCR Dock 2010[17] that were used for simulations with the OPLS and CHARMM protocols.

			RMSD/Å [a]		
**Name**	**Submission name**	**Model #**	**TM**	**EL2**	**LIG**
3pbl	PDB: 3PBL	-	-	-	-
M-01	5084	3	1.36	2.92	0.94
M-02	3646	4	1.42	3.46	1.10
M-03	3041	5	1.35	12.5	1.78
M-04	1813	3	1.49	4.33	1.79
M-05	3801	3	1.36	3.50	2.07
M-06	8004	1	1.91	4.42	2.13
M-07	1180	3	1.55	5.15	2.26
M-08	1135	1	2.30	4.21	2.27
M-09	4416	1	2.30	4.21	2.27
M-10	5334	2	1.90	3.13	2.32
M-11	2556	4	2.07	5.52	3.04
M-12	6006	2	1.62	5.73	3.08
M-13	1285	2	1.37	3.00	3.11
M-14	5508	1	1.50	4.05	3.31
M-15	3682	4	1.71	4.61	3.31
M-16	0460	1	1.49	3.39	3.47
M-17	7334	1	1.35	3.60	3.55
M-18	3532	5	2.41	3.93	3.56
M-19	4374	5	1.36	3.61	3.67
M-20	7141	2	1.51	4.97	3.74
M-21	2866	2	1.70	3.52	4.01
M-22	2632	1	2.34	4.80	4.02
M-23	2560	5	2.53	4.47	4.15
M-24	3682	1	1.72	4.64	4.18
M-25	0460	4	1.39	3.88	4.33
M-26	1285	3	1.36	2.96	4.50
M-27	1813	1	1.47	4.35	4.89
M-28	4374	3	1.42	3.13	5.84
M-29	1576	1	3.02	8.88	9.67
M-30	2364	2	15.9	21.3	12.5

[a] RMSD from D_3R crystal structure after alignment.

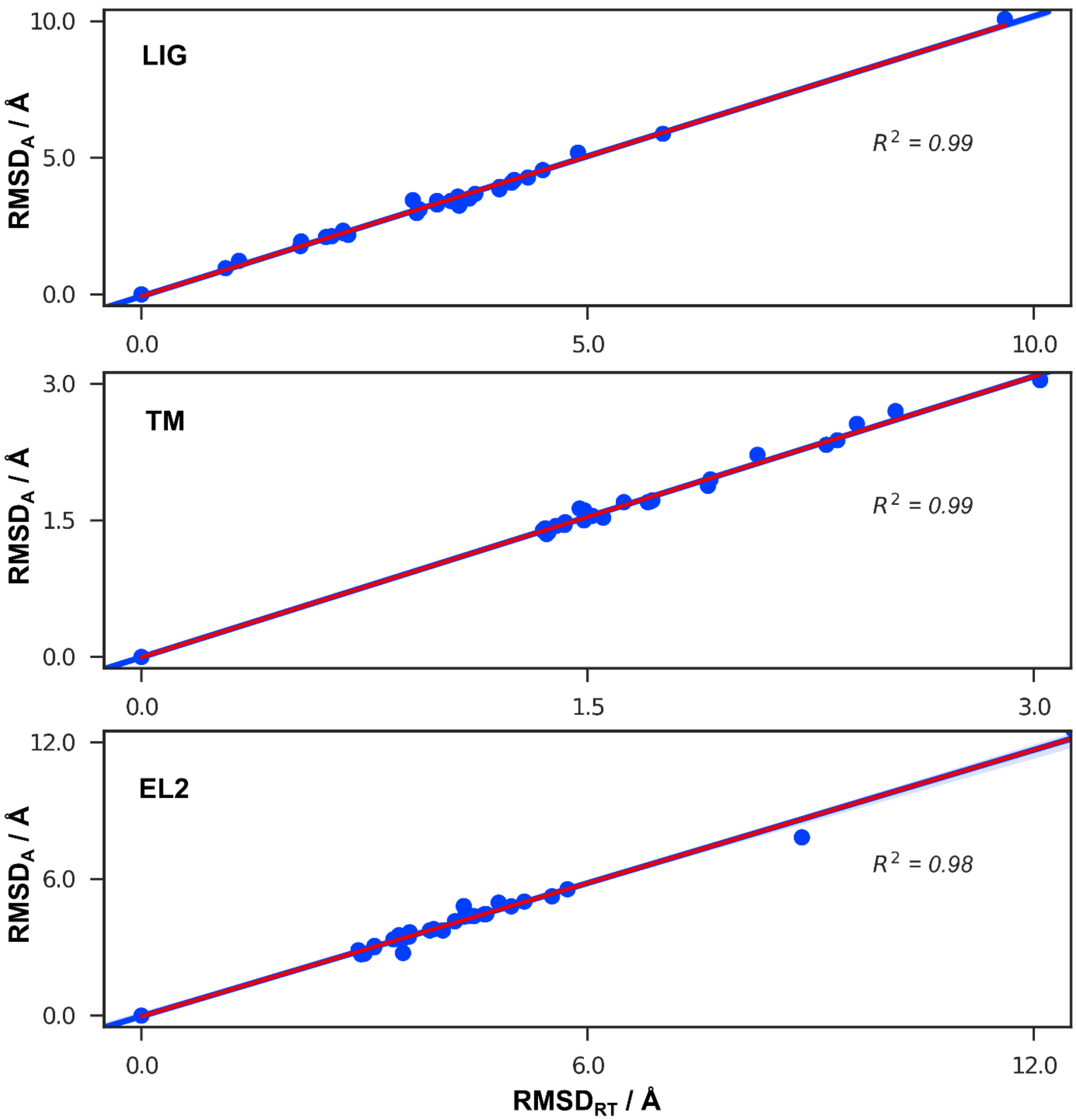

Fig A. Comparison of RMSD values calculated for the TM, EL2, and ligand (LIG) in this work using rotational/translational least squares fit of the TM region ($RMSD_{RT}$) and the GPCR Dock 2010 assessment [17] ($RMSD_A$). The model M-30 (2364_5) was treated as an outlier and is not included in the comparison.

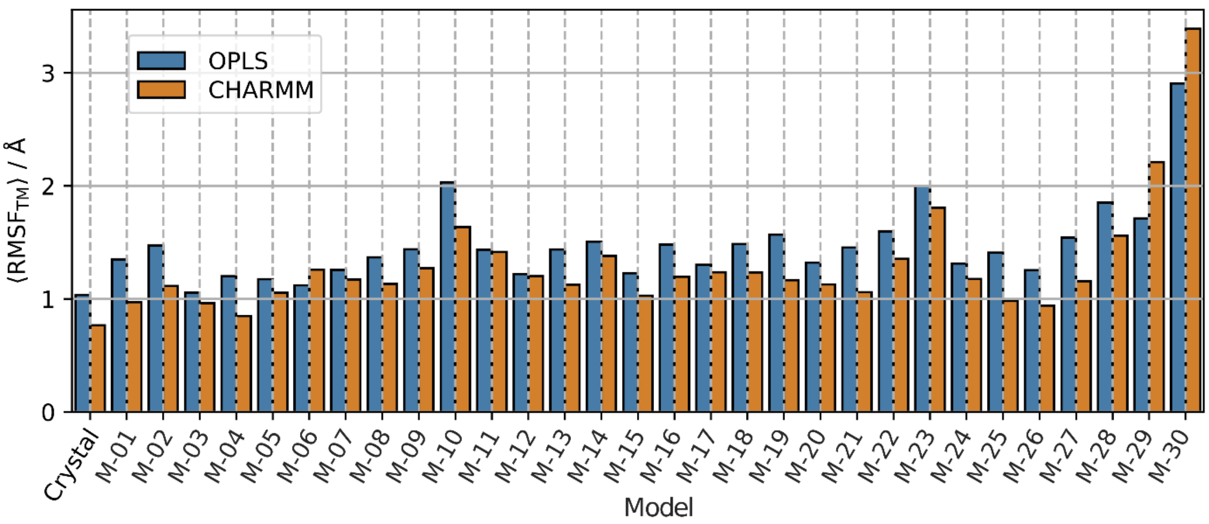

Fig B. Fluctuations in the TM region of D_3R crystal structure and models. Average RMSF values for TM region from the simulations performed with the OPLS (blue) and CHARMM (orange) protocols.

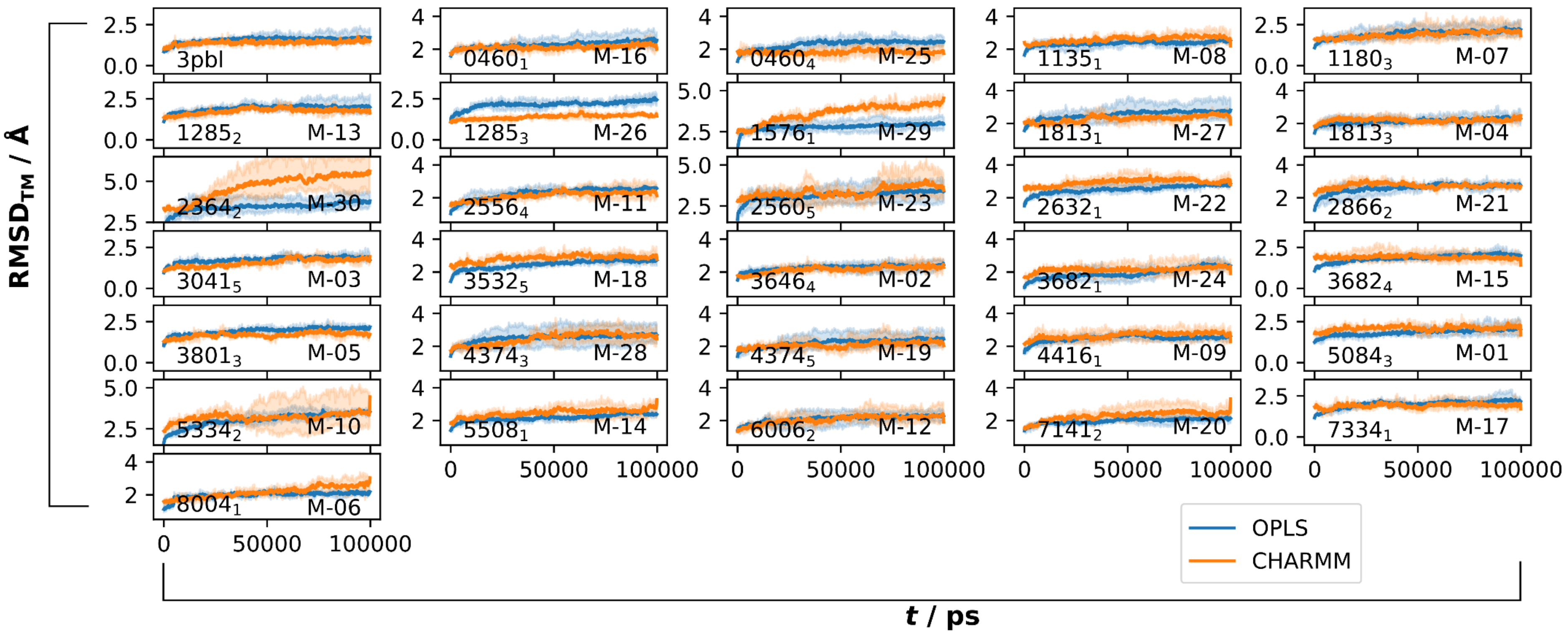

Fig C. $RMSD_{TM}$ calculated for each model and simulation protocol with the initial structures as reference. The three replicate simulations are averaged for OPLS (blue) and CHARMM (orange) at every snapshot in time. The standard error at 95% confidence interval is shown in paler colors.

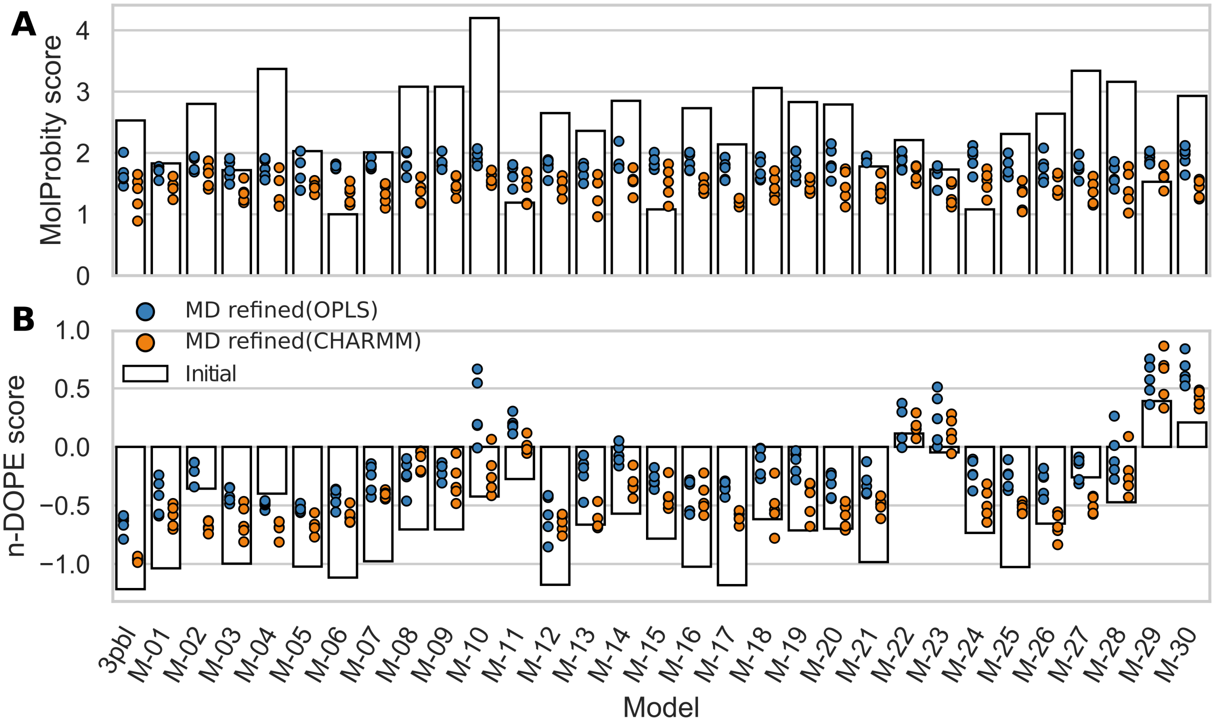

Fig D. Effect of MD refinement on protein quality scores. (A) MolProbity and (B) n-DOPE scores for the initial (bars) and MD refined (circles) structures using the OPLS (blue) and CHARMM (orange) protocols.

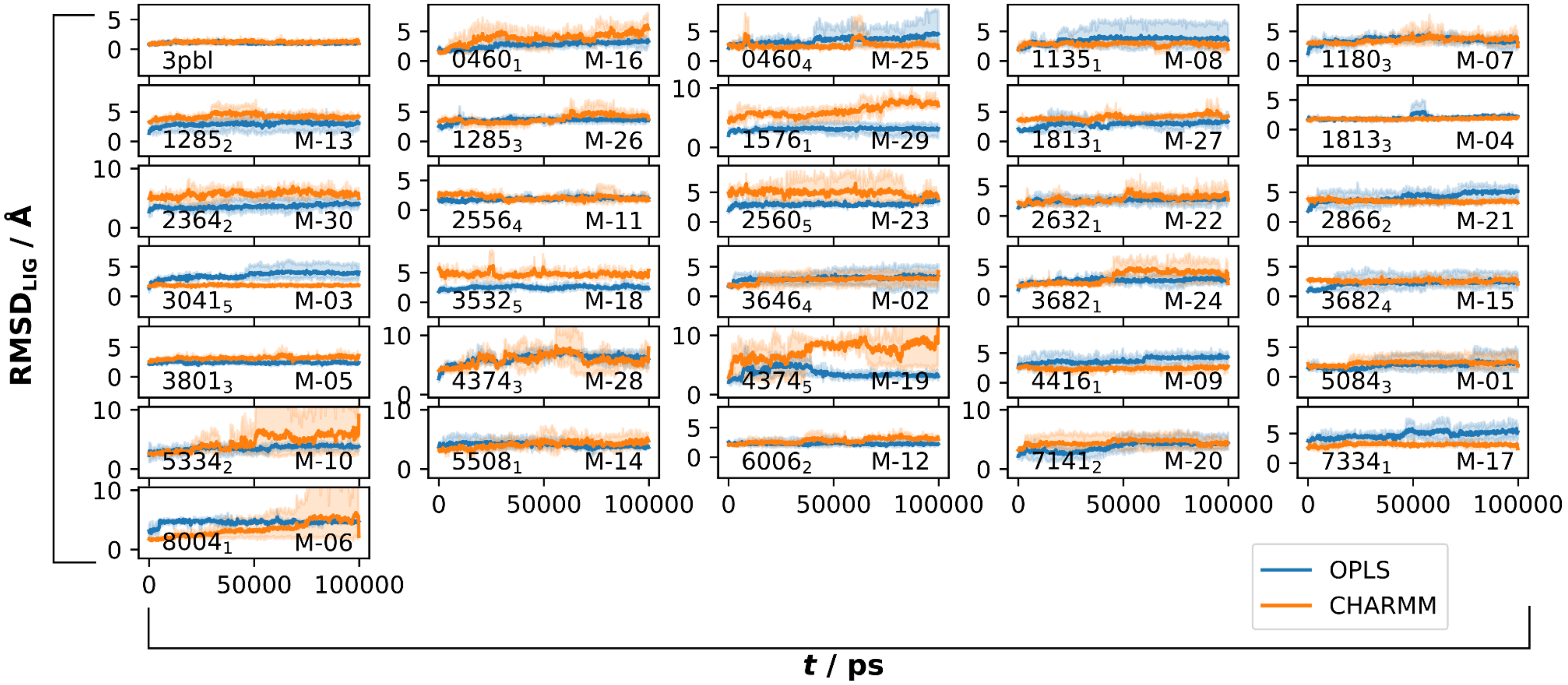

Fig E. $RMSD_{LIG}$ calculated for each model and simulation protocol with the initial structures as reference. The three replicate simulations are averaged for OPLS (blue) and CHARMM (orange) at every snapshot in time. The standard error at 95% confidence interval is shown in paler color.

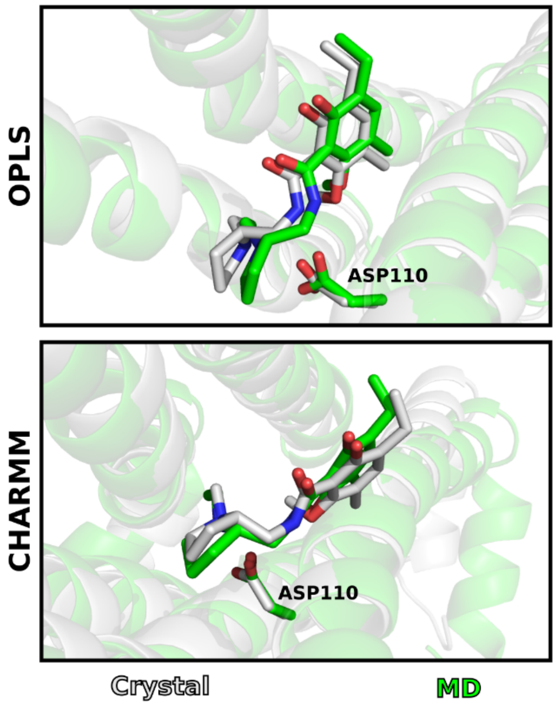

Fig F. Ligand poses from the simulations initiated from the crystal structure of D_3R. The receptor is shown as cartoons and the ligand in sticks. The best MD refined models and the crystal structure are colored green and grey, respectively.

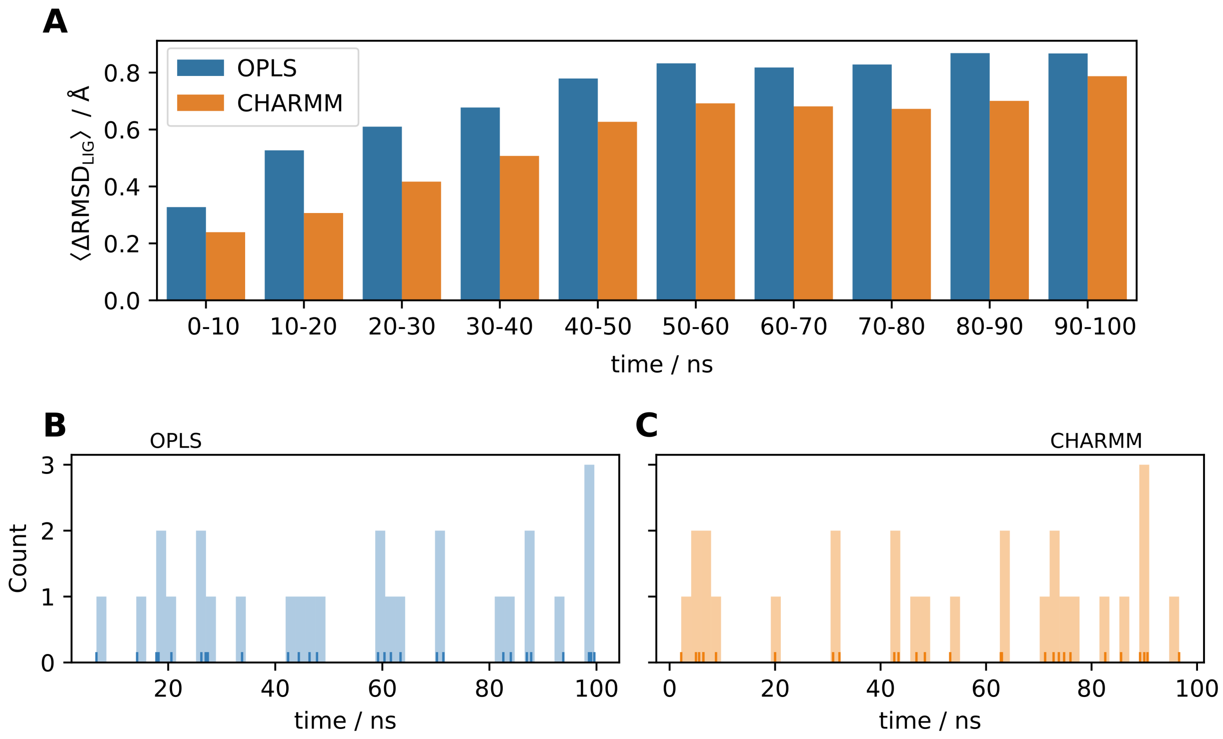

Fig G. Assessment of the effect of simulation length on $RMSD_{LIG}$. (A) The change in RMSD ($\Delta RMSD_{LIG}$) averaged in blocks of 10 ns over all three replicates of all MD refinement simulations. The distribution of the binding modes with the best $RMSD_{LIG}$ values for the (B) OPLS and (C) CHARMM protocols based on the centroids representing the five largest clusters for each model.

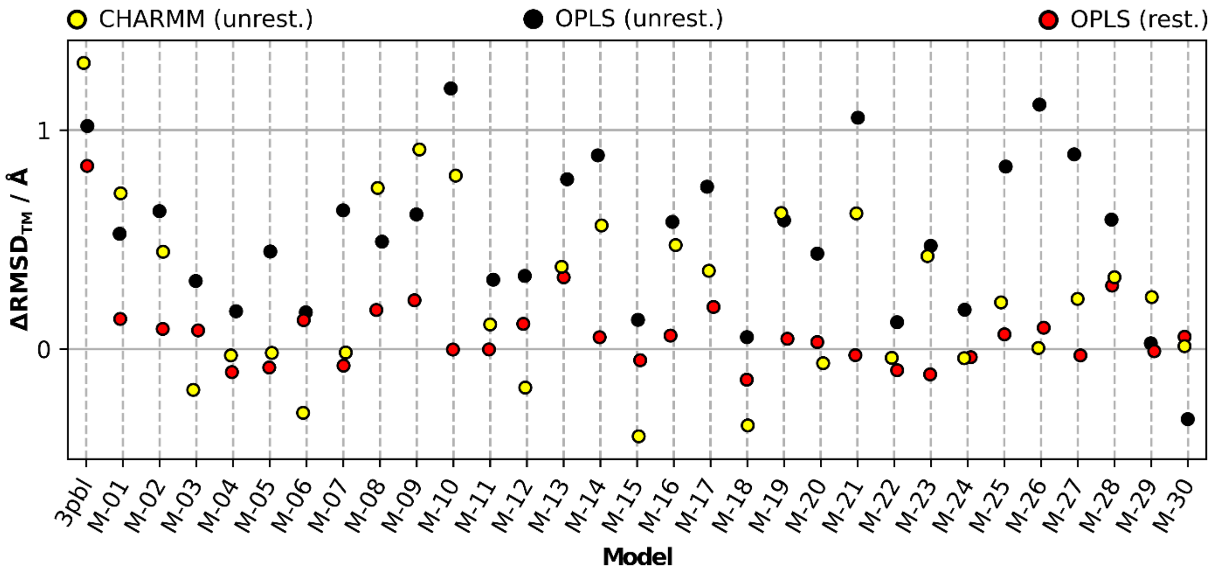

Fig H. Best ΔRMSD$_{TM}$ (difference in RMSD$_{TM}$ between the best MD refined and initial structure) from different simulation protocols. Data from unrestrained CHARMM (yellow), OPLS (black), and restrained OPLS (red) simulations are shown.

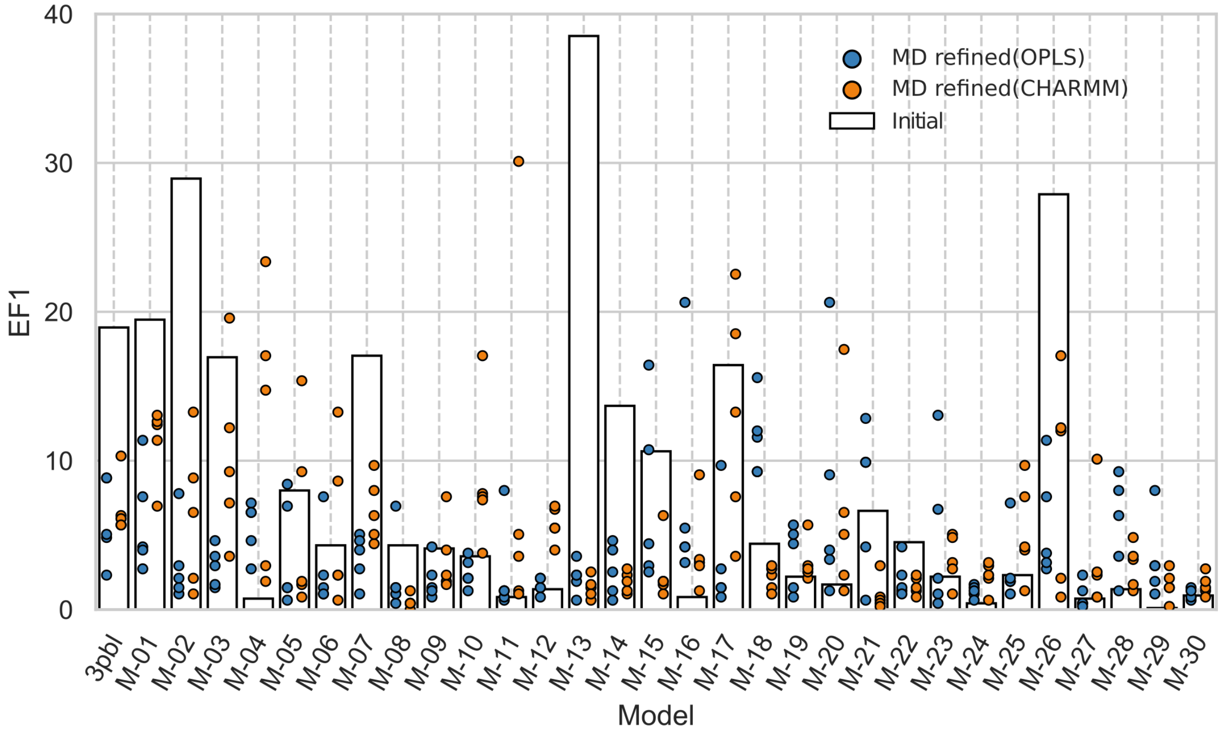

Fig I. Virtual screening performance of the initial and MD refined structures. EF1 results for the crystal structure and initial models are shown as bars. The EF1 values for the five MD refined models and crystal structures are shown as blue and orange circles for the OPLS and CHARMM protocols, respectively.

References

1. Abraham MJ, Murtola T, Schulz R, Páll S, Smith JC, Hess B, et al. GROMACS: High performance molecular simulations through multi-level parallelism from laptops to supercomputers. SoftwareX. 2015;1-2:19-25. doi: 10.1016/j.softx.2015.06.001.

2. Van Der Spoel D, Lindahl E, Hess B, Groenhof G, Mark AE, Berendsen HJC. GROMACS: Fast, flexible, and free. J Comput Chem. 2005;26:1701-18. doi: 10.1002/jcc.20291. PubMed PMID: 16211538.

3. Berendsen HJC, van der Spoel D, van Drunen R. GROMACS: A message-passing parallel molecular dynamics implementation. Comput Phys Commun. 1995;91:43-56. doi: 10.1016/0010-4655(95)00042-E.

4. Harvey MJ, Giupponi G, Fabritiis GD. ACEMD: Accelerating Biomolecular Dynamics in the Microsecond Time Scale. J Chem Theory Comput. 2009;5(6):1632-9. Epub 2009/06/09. doi: 10.1021/ct9000685. PubMed PMID: 26609855.

5. Kaminski GA, Friesner RA, Tirado-Rives J, Jorgensen WL. Evaluation and Reparametrization of the OPLS-AA Force Field for Proteins via Comparison with Accurate Quantum Chemical Calculations on Peptides†. J Phys Chem B. 2001;105(28):6474-87. doi: 10.1021/jp003919d.

6. Huang J, Rauscher S, Nawrocki G, Ran T, Feig M, de Groot BL, et al. CHARMM36m: an improved force field for folded and intrinsically disordered proteins. Nat Methods. 2017;14(1):71-3. Epub 2016/11/08. doi: 10.1038/nmeth.4067. PubMed PMID: 27819658; PubMed Central PMCID: PMC5199616.

7. Berger O, Edholm O, Jähnig F. Molecular dynamics simulations of a fluid bilayer of dipalmitoylphosphatidylcholine at full hydration, constant pressure, and constant temperature. Biophys J. 1997;72:2002-13. doi: 10.1016/S0006-3495(97)78845-3. PubMed PMID: 9129804.

8. Klauda JB, Venable RM, Freites JA, O'Connor JW, Tobias DJ, Mondragon-Ramirez C, et al. Update of the CHARMM All-Atom Additive Force Field for Lipids: Validation on Six Lipid Types. J Phys Chem B. 2010;114:7830-43. doi: 10.1021/jp101759q.

9. Schrödinger MacroModel v11. 11.3 ed: Scrödinger LLC., Portland, OR; 2015.

10. Vanommeslaeghe K, MacKerell AD, Jr. Automation of the CHARMM General Force Field (CGenFF) I: bond perception and atom typing. J Chem Inf Model. 2012;52(12):3144-54. Epub

2012/11/14. doi: 10.1021/ci300363c. PubMed PMID: 23146088; PubMed Central PMCID: PMC3528824.

11. Vanommeslaeghe K, Raman EP, MacKerell AD, Jr. Automation of the CHARMM General Force Field (CGenFF) II: assignment of bonded parameters and partial atomic charges. J Chem Inf Model. 2012;52(12):3155-68. Epub 2012/11/14. doi: 10.1021/ci3003649. PubMed PMID: 23145473; PubMed Central PMCID: PMC3528813.

12. Vanommeslaeghe K, Hatcher E, Acharya C, Kundu S, Zhong S, Shim J, et al. CHARMM general force field: A force field for drug-like molecules compatible with the CHARMM all-atom additive biological force fields. J Comput Chem. 2010;31(4):671-90. Epub 2009/07/04. doi: 10.1002/jcc.21367. PubMed PMID: 19575467; PubMed Central PMCID: PMC2888302.

13. Berendsen HJC, Grigera JR, Straatsma TP. The Missing Term in Effective Pair Potentials. J Phys Chem. 1987;91:6269-71. doi: 10.1021/j100308a038.

14. Jorgensen WL, Chandrasekhar J, Madura JD, Impey RW, Klein ML. Comparison of simple potential functions for simulating liquid water. J Chem Phys. 1983;79:926. doi: 10.1063/1.445869. PubMed PMID: 25246403.

15. Parrinello M, Rahman A. Polymorphic transitions in single crystals: A new molecular dynamics method. J Appl Phys. 1981;52:7182. doi: 10.1063/1.328693.

16. Bussi G, Donadio D, Parrinello M. Canonical sampling through velocity rescaling. J Chem Phys. 2007;126:014101. doi: 10.1063/1.2408420. PubMed PMID: 17212484.

17. Kufareva I, Rueda M, Katritch V, Stevens RC, Abagyan R. Status of GPCR Modeling and Docking as Reflected by Community-wide GPCR Dock 2010 Assessment. Structure. 2011;19:1108-26. doi: 10.1016/j.str.2011.05.012.
